# Demographic, socioeconomic and life-course risk factors for internalized weight stigma in adulthood: evidence from an English birth cohort study

**DOI:** 10.1016/j.lanepe.2024.100895

**Published:** 2024-04-15

**Authors:** Amanda M. Hughes, Stuart W. Flint, Ken Clare, Antonis A. Kousoulis, Emily R. Rothwell, Helen Bould, Laura D. Howe

**Affiliations:** a MRC Epidemiology Unit, Population Health Sciences, Bristol Medical School, University of Bristol, Bristol, UK; b School of Psychology, University of Leeds, Leeds, UK; c Scaled Insights, Nexus, University of Leeds, Leeds, UK; d Leeds Beckett University, Leeds, UK; e Obesity UK, Halifax, UK; f European Coalition for People Living with Obesity, Dublin, Ireland; g Global Mental Health Action Network, London, UK; h Our Public Health, London, UK; i Greater Manchester Mental Health NHS Foundation Trust, Manchester, UK; j Centre for Academic Mental Health, Population Health Sciences, Bristol Medical School, University of Bristol, Bristol, UK; k Gloucestershire Health and Care NHS Foundation Trust, Gloucestershire, UK; l Population Health Sciences, Bristol Medical School, University of Bristol, UK

**Keywords:** Weight stigma, Weight bias, ALSPAC, Bullying, Longitudinal, Inequalities

## Abstract

**Background:**

Obesity is highly stigmatized, with negative obesity-related stereotypes widespread across society. Internalized weight stigma (IWS) is linked to negative outcomes including poor mental health and disordered eating. Previous evidence examining population groups at higher risk of experiencing IWS comes from small, nonrepresentative samples. Here, we re-assess previously reported associations of IWS with demographic, socioeconomic, and wider social factors in a large general population birth cohort study for the first time.

**Methods:**

In the Avon Longitudinal Study of Parents and Children (ALSPAC), we explored differences in IWS at age 31 years by sex, ethnicity, socioeconomic factors, sexual orientation, and family and wider social influences, using confounder-adjusted multivariable regression.

**Findings:**

In models adjusted for potential confounders and BMI in childhood, adolescence, and adulthood (N = 4060), IWS was higher for females (standardized beta: 0.56, 95% CI: 0.50, 0.61), sexual minorities (0.17 S.D. higher, 95% CI: 0.09, 0.24), and less socioeconomically advantaged individuals (e.g., 0.16 S.D. higher (95% CI: 0.08, 0.24) for participants whose mothers had minimum or no qualifications, compared to a university degree). The social environment during adolescence and young adulthood was important: IWS was higher for people who at age 13 years felt pressure to lose weight from family (by 0.13 S.D., 95% CI: 0.03, 0.23), and the media (by 0.17, 95% CI: 0.10, 0.25), or had experienced bullying (e.g., 0.25 S.D., 95% CI: 0.17, 0.33 for bullying at age 23 years).

**Interpretation:**

Internalized weight stigma differs substantially between demographic groups. Risk is elevated for females, sexual minorities, and socioeconomically disadvantaged adults, and this is not explained by differences in BMI. Pressure to lose weight from family and the media in adolescence may have long-lasting effects on IWS.

**Funding:**

The 10.13039/501100000269ESRC, 10.13039/501100000265MRC, 10.13039/501100000272NIHR, and 10.13039/100010269Wellcome Trust.

**Research in context**

**Evidence before this study**

Previous evidence on internalized weight stigma (IWS) has largely come from non-representative sample populations. Longitudinal evidence is lacking, and no study has explored risk factors for IWS using birth cohort data. In January 2024 we searched PubMed for articles containing “internalized weight stigma” or “internalized weight bias” and “longitudinal” or “cohort” in the title or abstract, with no date restrictions. Of 11 longitudinal studies, 5 were of university students, and four of people undergoing bariatric surgery or enrolled in weight-management programmes. The longest follow-up was 16 months.

**Added value of this study**

This is the first study to explore risk factors across the life-course related to IWS among adults from the general population. The study examined relationships within a general population birth cohort study, exploring the impact of under-examined factors including socioeconomic disadvantage, and IWS among male and female sexual minorities. Using data collected over a 32-year period, we identify risk factors across the life-course, whilst minimising influence of recall bias.

**Implications of all the available evidence**

Females, socioeconomically disadvantaged people, and male and female sexual minorities are at greater risk of IWS. The family environment in adolescence, bullying, and feeling under pressure to lose weight from the media may have long-lasting impacts on adult IWS and may be fruitful targets for intervention to reduce IWS and its consequences.

## Introduction

In England, approximately 26% of adults and 23% of children are living with obesity.^1^ Weight-related stigma and discrimination are reported across society, and these experiences are consistently associated with impaired mental health^2^ and lower quality of life.^3^ People who report experiences of weight-related stigma or discrimination are at greater risk^4^ of internalized weight stigma (IWS), usually defined as agreement with and self-application of negative weight stereotypes, often leading to reduced self-worth.^5^ Among people living with obesity, IWS is linked to disordered eating,^6^ worse mental health,^7^ and healthcare avoidance.^8^ People with a higher body mass index (BMI) report more experiences of weight-related stigma^9^ and greater IWS.^10^ However, IWS can also affect people within the recommended and underweight body mass index (BMI) categories, where it predicts disordered eating and drive for thinness,^11^ making it relevant for mental health across the body weight range.

A growing body of work suggests that, independently of a person's weight, other factors are likely to influence IWS. Most studies find greater IWS among women compared to men,^10^^,^^12^^,^^13^ and a representative German study reported a link with socioeconomic disadvantage, as measured by either education or income.^10^ Some US studies report higher IWS for white compared to African American participants,^4^^,^^14^ but how IWS relates to ethnicity outside of the US in unknown. Likewise, differences by sexual orientation remain under-researched; existing studies have reported higher IWS among sexual minority men compared to heterosexual men,^15^, ^16^, ^17^ but no difference by sexual orientation for women.^16^^,^^17^ Stigmatizing experiences from family members and at work have been cross-sectionally linked with IWS,^18^ and longitudinal evidence suggests that weight-based teasing in adolescence by both family and peers can have long-lasting consequences for disordered eating and body image.^19^^,^^20^ However, research examining IWS has overwhelmingly been cross-sectional,^21^ precluding investigation of risk-factors over the life-course without risk of recall bias. Furthermore, evidence has largely come from small, nonrepresentative sample populations, such as university students or weight support groups.^21^ This limits the generalizability of findings, and the extent to which causal processes can be explored.^22^ Finally, most evidence has come from the US, again limiting generalizability, since contextual factors including a country's prevalence of obesity are likely to shape both the nature and extent of weight stigma.^23^

The aim of this study was to re-assess previously reported associations of IWS with demographic, socioeconomic, and wider social factors in a large general population study from Europe, and to explore to what extent associations are explained by differences in current and former BMI. For the first time, we describe risk factors for IWS among 4060 adults in a general population, longitudinal birth cohort study from England. We examine the role of sex, ethnicity, sexual orientation, socioeconomic factors in childhood and young adulthood, and family, peer and wider social influences during adolescence and early adulthood, exploring whether associations with these factors are independent of BMI in childhood, adolescence and early adulthood.

## Methods

### Study participants

The Avon Longitudinal Study of Parents and Children (ALSPAC) began as a pregnancy study of expectant mothers living in or around Bristol (UK) with expected delivery dates between 1/4/1991 and 31/12/1992.^24^ From the initial 14,541 pregnancies, 13,988 children were alive after 1 year. Mothers, partners, and children have been followed up through regular questionnaires and clinics. We drew on data from clinics held when participants were aged 7, 8, 9, 10, 11.5, 12.5, 13.5, 15.5, 17.5 and 24 years old, questionnaires completed at age 13 years and annually from ages 21–31 years, and questionnaires completed by their mothers during pregnancy and the participant's early childhood. The initial sample was broadly representative of the UK population in the 1991 census, with underrepresentation of single parent families, those living in rented accommodation and some ethnic minorities (see Supplementary Methods). This analysis was restricted to the 4060 participants who completed the age 31 years questionnaire in 2022.

### Measures

#### Internalized weight stigma

Participants completed the Modified Weight Bias Internalization Scale (WBIS-M)^5^ for the first time at approximately 31 years of age. The WBIS-M, which is suitable for people of any weight status, measures self-attribution of obesity-related stereotypes and the degree of self-evaluation based on weight. Participants rate their agreement from 1 (“does not apply to me at all”) to 7 (“applies to me perfectly”) with 11 statements such as “I am less attractive than most other people because of my weight” and “I hate myself for my weight” (see Supplementary Methods). Following recent practice,^4^^,^^12^ a summary index was constructed by adding 10 of the 11 items, given psychometric data supporting removal of item 1 from the original scale.^10^ The 10 items showed very good internal consistency (Cronbach's alpha = 0.96, McDonald's omega = 0.96). In a sensitivity analysis, an index considering all 11 items (Cronbach's alpha = 0.93, McDonald's omega = 0.95) was used. Both indexes were included as continuous variables, standardized to have a mean of zero and standard deviation of one for analysis.

#### Demographic and socioeconomic factors

Participants' sex as recorded at birth was coded as male or female. Participant ethnicity was based on self-described ethnicity at age 28 years. For 13.3% of participants this information was missing, and a report by the mother of the young person's ethnicity was used instead. Due to relatively few participants from ethnic minority backgrounds in the sample, this was dichotomized as white (95.8% of participants) or any other ethnicity (4.2% of participants). Socioeconomic position in childhood was based on educational qualifications of the participants' mother. This was categorized into three groups: as qualifications usually taken at age 16 years (GCSEs/O-levels) or less, qualifications usually taken at age 18 years (A-levels, vocational qualifications), or a university degree. For participants' own socioeconomic position in adulthood, we firstly considered whether they had attended university by age 30 years (coded yes/no). This information was available for 85.7% of participants; where it was not, we used a similar report from age 26 years (6.6% of participants) and reports of having graduated at 22, 23, 24, and 25 years (1.7% of participants). Second, we combined information from age 21, 22, 23, 25, 27, and 29 years to derive the number of occasions on which they had been NEET (not in education, employment, or training) between age 21 and 29 years. This was categorized into three groups for analysis (never, once, or twice or more). Participants reported their sexual orientation at ages 15, 23, and 31 years. We used the age 31 report where available (96.6% of participants), and the latest available report otherwise. Due to small cell sizes, this was dichotomized as heterosexual/straight (85.7%) or any other orientation (14.3%, hereafter “sexual minorities”), including 1.7% of participants who answered, “don't know”.

#### BMI from age 7–24 years

Repeated measures of participants’ height and weight are available from regular research clinics which began when participants were aged 7 years. Based on an a priori decision, we consider BMI at six points during development: mid childhood (age 7 years), later childhood (age 10 years), early adolescence (age 12.5 years), mid adolescence (age 15.5 years), later adolescence (age 17.5 years), and early adulthood (age 24 years). BMI in standard units (kg/m^2^) is not an appropriate measurement for children and adolescents who are still growing. For this reason, at ages 7, 10, 12.5 and 15.5 years, height and weight were used to calculate gender- and age-specific BMI z-scores standardized to the 1990 UK Growth Reference.^25^ A z-score of 0 equates to the 50th percentile, and a z-score of ±1.0 plots at the 15th or 85th percentiles, for children of a specified age and gender. At ages 17.5 and 24 years BMI is included in standard units (kg/m^2^), and associations expressed per 5 kg/m^2^, equivalent to the width of a BMI category (e.g., 25.0–29.9 kg/m^2^).

#### Family, peer, and wider social influences in adolescence

At age 13 years, adolescents reported the following.

1. How often in the past year their mother or father had made a comment about their weight or the amount they were eating, that made them feel bad (never/sometimes/often/always);
2. To what extent family members teased them about their weight or body shape (not a lot/a little/quite a lot/a lot);
3. To what extent people at school teased them about their weight or body shape (not a lot/a little/quite a lot/a lot);
4. To what extent they had felt pressure to lose weight from their family, friends, people they had dated, and the media (not at all/a little/quite a lot/a lot).

Due to small cell sizes, binary variables (coded never/ever) were derived for each of these variables.

Young people reported bullying victimization (being the target of bullying) at ages 8, 10, 12.5, 17.5 and 23 years. At age 8, 10, and 12.5 years, clinic assessments explored direct bullying (e.g., being called nasty names, threatened, or hit) and relational bullying (e.g., peers not spending time with the young person to upset them, or telling lies about them).^26^ At the age 17.5 years clinic, participants were also asked about cyber bullying. At age 23 years, young people reported in a questionnaire direct, relational, and cyber bullying in the past 6 months. Binary variables for any bullying victimization were derived for each timepoint, and associations with bullying types (direct/relational/cyber) explored as secondary analyses.

### Statistical analysis

Among participants who completed the age 31 questionnaire (N = 4060), we used multiple imputation by chained equations (m = 50) to impute missing values in all variables (details are provided in Supplementary Methods). The proportion of data imputed for most variables was relatively low (0.5% for the WBIS-M at 31 years, and ∼26% for factors reported at age 13 years) but higher (44.7%) for bullying at age 17.5 years. The percentage of imputed data for each variable is shown in Table S3. Associations between IWS and risk factors were explored using multivariable linear regression. We adjusted for appropriate confounders for each separate risk factor, identified as factors which temporally preceded, and could plausibly influence, both the risk factor and IWS at age 31 years (details in Supplementary Methods and Table S1). For all risk factors these included sex, ethnicity, and maternal educational qualifications. For BMI at age 24 years, this included BMI at all previous timepoints. For family and wider social factors reported at age 13 years, we explored further adjustment for other family and wider social factors reported at the same time. In models including bullying victimization, we explored further adjustment for BMI at the time and bullying victimization at earlier and later timepoints. For bullying victimization at age 23 years, the only BMI measurement close to the bullying report was shortly afterward (at age 24 years) and this was used instead. In addition to adjusting for potential confounders, for each exposure we include a model adjusting for BMI at age 24 years, to see whether associations are independent of BMI in adulthood. We ran sex-stratified analyses based on sex-stratified imputation models. Due to small cell sizes, it was not possible to examine sex-stratified associations with NEET history, or with distinct types of bullying. In sensitivity analyses, models were run using the 11-item version of the WBIS-M, and main models re-run using only complete-case data. All analysis was performed in STATA v17.

### Role of the funding source

The funders had no role in the conceptualization or design of the study, data collection, analysis, interpretation, writing of the manuscript, or the decision to publish.

## Results

### Descriptive characteristics of the sample

Characteristics of the analytic sample (N = 4060) based on imputed data are shown in Table 1. Compared to the rest of the ALSPAC cohort (i.e., those who did not complete the age 31 years questionnaire), participants in the analytic sample had a slightly lower BMI at most timepoints. At age 7 years, their mean z-score was 0.06 lower (95% CI: 0.01, 0.11, p = 0.01), and at age 24 years, their BMI was 0.35 kg/m^2^ lower (95% CI: 0.01, 0.69, p = 0.04). Retained participants were more likely to be female (66.4%, vs 42.4% of excluded participants) and white (95.9% vs 94.4%), tended to have mothers with higher qualifications (e.g., 19.1% vs 10.3% with a degree), and were more likely to identify as heterosexual (85.8% vs 79.3%) (for all differences, p < 0.001). Participants in the analytic sample were not more likely to have attended university, and did not differ on NEET history. A full comparison of the analytic sample and the rest of the ALSPAC sample based on complete-case data is presented in Table S2.

**Table 1:** Descriptive Characteristics of Analytic Sample, based on imputed data. N = 4060.^a^

Continuous variables	Mean	SD^b^
WBIS-M Score^c^	20.1	17.0
BMI^d^ z-score at age 7^e^ years	0.1	1.0
BMI z-score at age 10^e^ years	0.3	1.2
BMI z-score at age 12.5^e^ years	0.4	1.2
BMI z-score at age 15.5^e^ years	0.4	1.1
BMI (kg/m^2^) at age 17.5 years	23.0	4.4
BMI (kg/m^2^) at age 24 years	25.1	5.3
**Categorical variables**	**Category**	**%**

Sex	Female	66.4
	Male	33.6
Ethnicity	White	95.8
	Any other ethnicity	4.2
Mother's educational qualifications	GCSEs/O-levels/no qualifications	47.6
	A-levels/vocational qualification	33.8
	University degree	18.6
Attended university by age 30 years	No	33.4
	Yes	66.6
Occasions not in education, employment, or training (NEET) between age 21 and age 29 years	Never	74.1
	Once	17.7
	Twice or more	8.3
Sexual orientation	Heterosexual	84.6
	Any other orientation	15.4
Parents make negative comments about young person's weight^f^	No	72.1
	Yes	27.9
Teased by family about weight/shape^f^	No	75.4
	Yes	24.6
Teased at school about weight/shape^f^	No	76.2
	Yes	23.8
Pressure to lose weight from friends^f^	No	76.2
	Yes	23.8
Pressure to lose weight from family^f^	No	76.9
	Yes	23.1
Pressure to lose weight from girls/boys the young person has gone out with^f^	No	86.2
	Yes	13.8
Pressure to lose weight from media^f^	No	64.5
	Yes	35.0
Age 8 years: Bullying victimization	No	62.6
	Yes	37.4
Age 10 years: Bullying victimization	No	77.0
	Yes	23.0
Age 12.5 years: Bullying victimization	No	45.4
	Yes	54.6
Age 17.5 years: Bullying victimization	No	68.4
	Yes	31.6
Age 23 years: Bullying victimization	No	80.1
	Yes	19.9

a Descriptive characteristics based on complete-case data are shown in Table S1.

b SD = standard deviation.

c WBIS-M: Modified Internalized Weight Bias Questionnaire, range = 0–60.

d BMI = Body Mass Index.

e Z-scores are age- and sex-specific, based on the 1990 British growth references curves. A z-score of 0 equates to 50th percentile, and a z-score of ±1.0 plots at the 15th or 85th percentiles, respectively.

f Reported at age 13 years.

### Sociodemographic risk factors

**Fig. 1:**
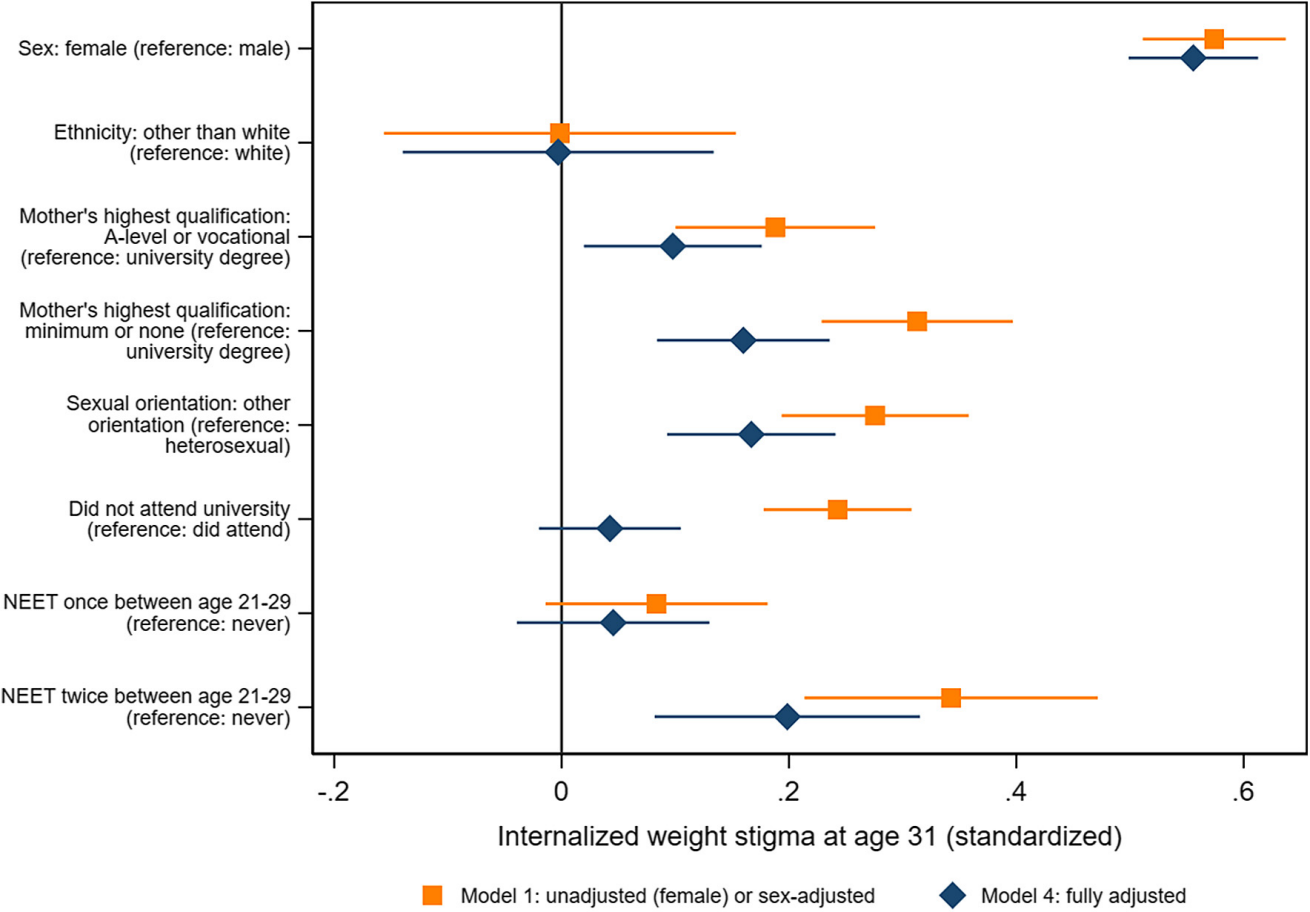
**Sociodemographic predictors of Internalized weight stigma at age 31 years, with and without adjusting for potential confounders and BMI across the life course**. **Note:** Covariates in fully adjusted models: Sex model: BMI at 7–24 years. Ethnicity model: sex, BMI at 7–24 years. Maternal qualifications model: sex, ethnicity, BMI at 7–24 years. Sexual orientation model: sex, ethnicity, maternal qualifications, BMI at 7–24 years. University attendance and NEET history models: sex, ethnicity, maternal qualifications, sexual orientation, BMI at 7–24 years.

Demographic differences in IWS are shown in Fig. 1 and Table S4. IWS was substantially and consistently higher among females than males (0.56 S.D., 95% CI: 0.50, 0.61, p < 0.001 with full adjustment). In contrast, there was little evidence of differences by ethnicity (0.00, −0.14, 0.13, p = 0.97). IWS was socially patterned by maternal qualification level: at all levels of adjustment, young people born to mothers with fewer qualifications had higher IWS. These differences attenuated with adjustment for the young person's adult BMI, but in fully-adjusted models, participants whose mothers had no or minimum qualifications had 0.16 S.D. higher IWS (95% CI: 0.08, 0.24, p < 0.001) than those whose mothers had a university degree. Sexual minority participants had elevated IWS, and this was not explained by other demographic factors or BMI at any point in development: in fully adjusted models, IWS was 0.17 S.D. higher (95% CI: 0.09, 0.24, p < 0.001) compared to heterosexual participants. Participants who had not attended university had higher IWS than those who had, but successive adjustment (Table S3) showed that this was largely explained by differences in adult BMI (0.04 S.D., 95% CI: −0.04, 0.13, p = 0.29, in fully-adjusted models). In contrast, people who had been not in education, employment, or training twice or more during their twenties had higher IWS after full adjustment (0.20 S.D., 95% CI: 0.08, 0.32, p = 0.001).

### BMI from age 7–24 years

**Fig. 2:**
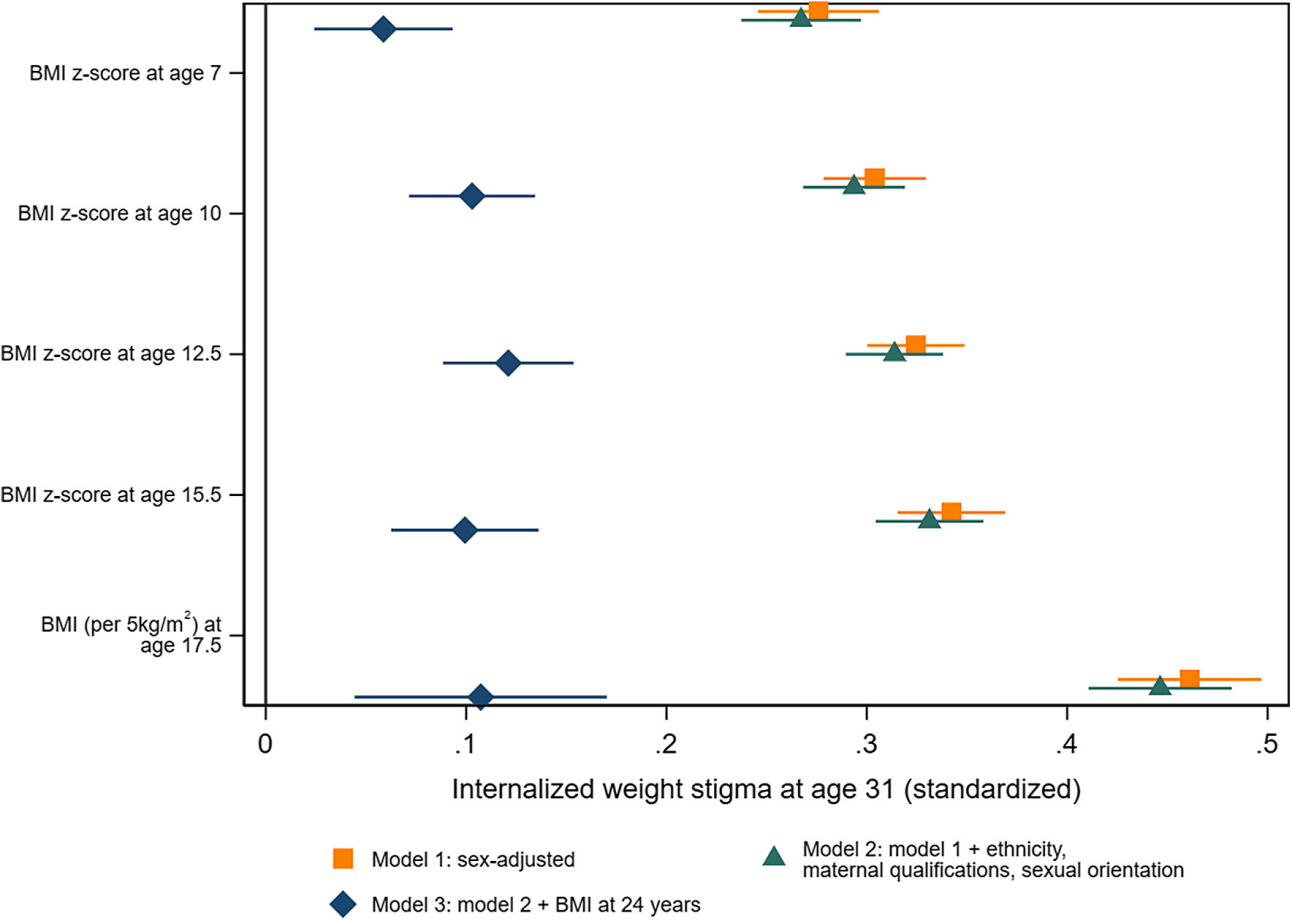
**BMI at ages 7, 10, 12.5, 15.5, and 17.5 years and internalized weight stigma (IWS) at age 31 years, with and without adjusting for sociodemographic factors and BMI at age 24 years**.

**Fig. 3:**
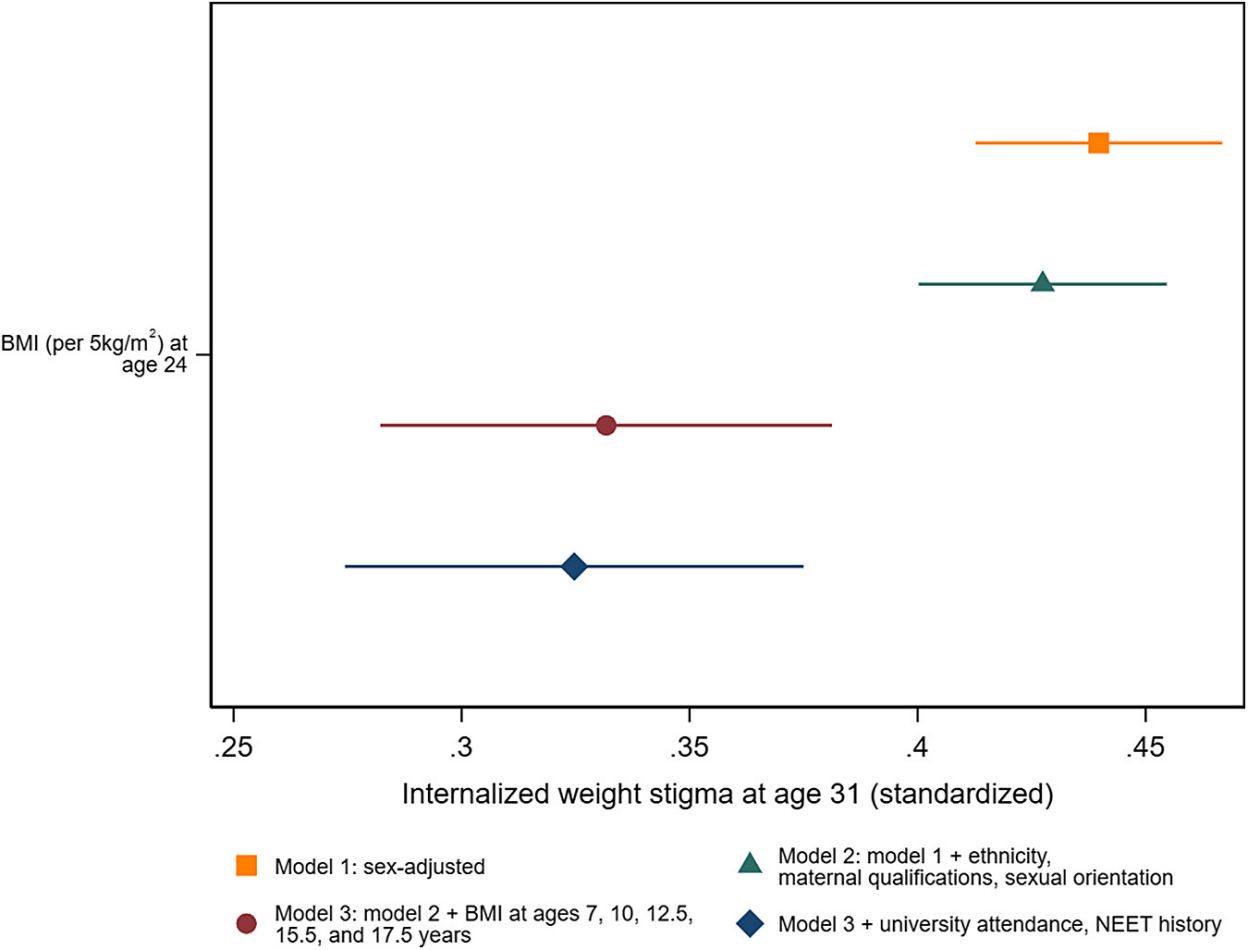
**BMI at ages 24 years and internalized weight stigma (IWS) at age 31 years, with and without adjusting for potential confounders**.

As shown in Fig. 2 and Table S5, higher BMI in mid childhood (age 7 years), late childhood (age 10 years), early adolescence (age 12.5 years), mid adolescence (15.5 years), late adolescence (age 17.5 years) and early adulthood (age 24 years, Fig. 3), were clearly and independently associated with IWS at age 31 years. Adjustment for earlier and later demographic factors made little difference to estimates. Consistent with BMI tracking over time,^27^ coefficients for earlier BMI attenuated with adjustment for BMI at age 24 years, for example from 0.46 (95% CI: 0.43, 0.50, p < 0.001 per 5 kg/m^2^) to 0.11 (0.04, 0.17, p = 0.001) for BMI at age 17.5 years. Nevertheless, BMI at all points considered robustly predicted IWS at age 31 years, independent of BMI in adulthood and confounders.

### Family, peer, and wider social influences at age 13 years

**Fig. 4:**
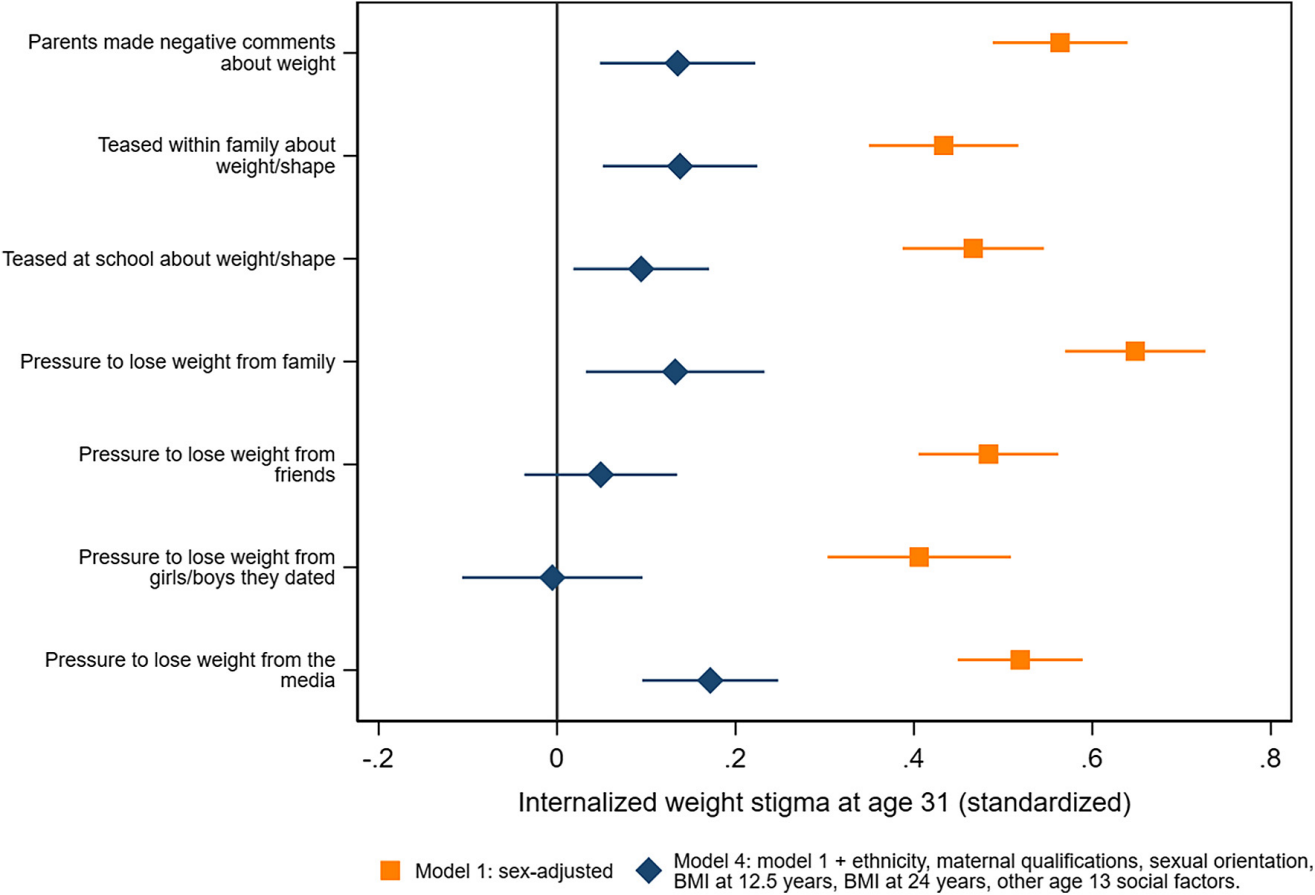
**Family, peer and wider social influences at age 13 years and internalized weight stigma (IWS) at age 31 years**.

In sex-adjusted models, IWS was higher for young people who at age 13 years reported any of the following: weight-related teasing from their family, weight-related teasing at school, negative weight-based comments from parents, and feeling under pressure to lose weight from family, friends, people they had dated, and the media (Fig. 4, Table S6). The strongest associations were for negative weight-related comments from parents (0.56 S.D., 95% CI: 0.49, 0.64, p < 0.001), and feeling under pressure to lose weight from family (0.65 S.D., 95% CI: 0.57, 0.73, p < 0.001), and the media (0.52 S.D., 95% CI: 0.45, 0.59, p < 0.001). Adjustment for ethnicity, maternal qualifications and sexual orientation made little difference to estimates, but associations did attenuate with adjustment for BMI at 12.5 years, other social influences at age 13 years, and adult BMI. Nevertheless, in fully-adjusted models, all age 13 factors apart from pressure to lose weight from friends or people the young person had dated independently predicted IWS at 31 years.

### Bullying victimization at ages 8, 10, 12.5, 17 and 23 years

**Fig. 5:**
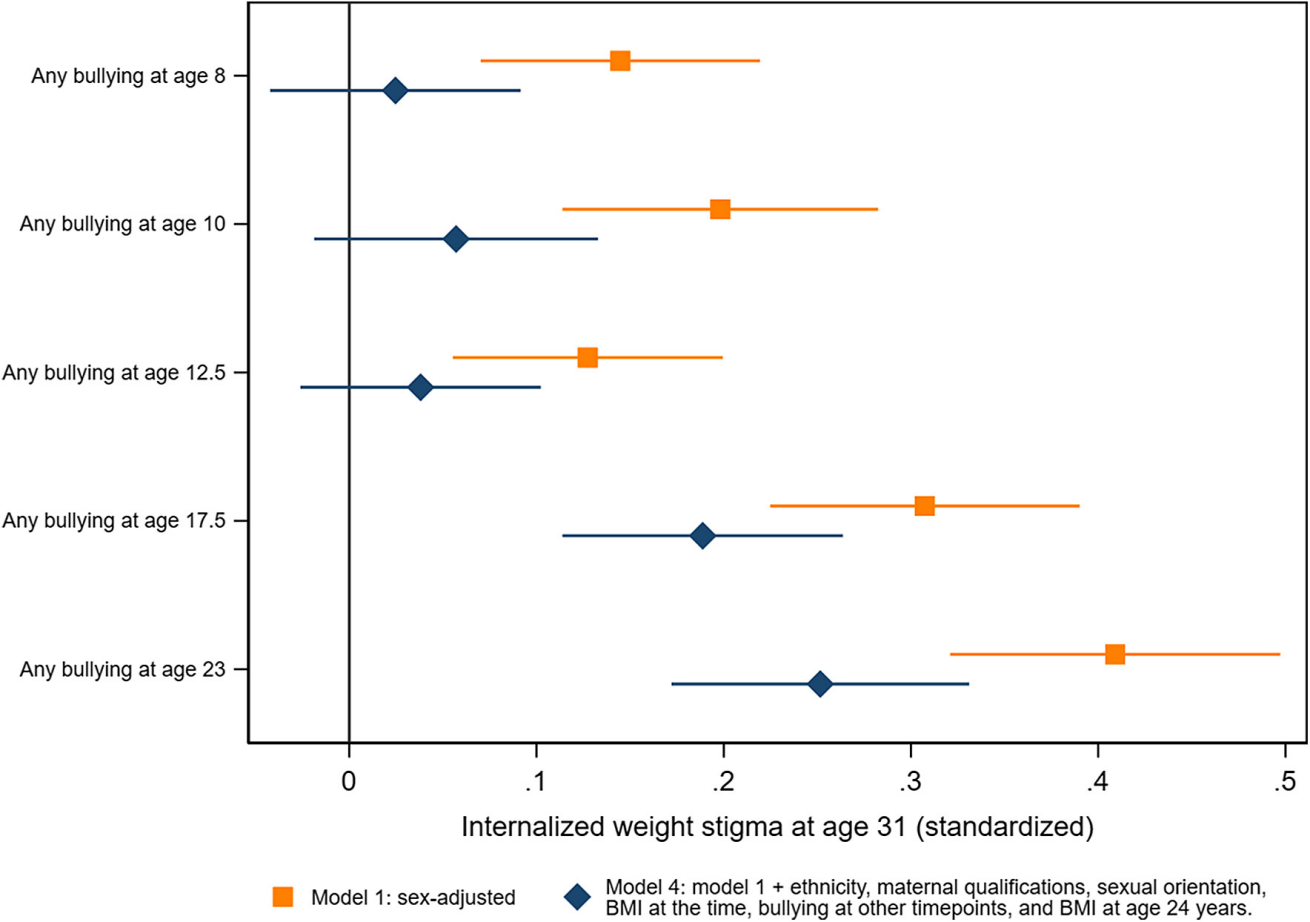
**Bullying victimization at ages 8, 10, 12.5, 17.5 and 23 years and internalized weight stigma (IWS) at age 31 years**.

In sex-adjusted models, bullying victimization at every timepoint was associated with IWS at age 31 years (Fig. 5, Table S7), but associations were stronger for more recent bullying victimization (0.41, 95% CI: 0.32, 0.50, p < 0.001 for bullying at age 23 years, and 0.14, 95% CI: 0.07, 0.22, p < 0.001 for bullying at age 8 years). Adjusting for demographic confounders, BMI at the time, bullying at other timepoints, and BMI at age 24 years attenuated estimates. In fully-adjusted models, bullying at ages 8, 10 and 12.5 years no longer independently predicted IWS at age 31 years, but bullying at ages 17.5 and 23 years did (0.19, 95% CI: 0.11, 0.26, p < 0.001, and 0.25, 95% CI: 0.17, 0.33, p < 0.001). Associations with direct, relational and cyber bullying were qualitatively similar, but estimates less precise (Figure S2, Table S6).

### Sex-stratified and additional models

In sex-stratified analysis (Tables S8–S11), smaller sample sizes led to less precise estimates, especially for males (N = 1363). Most associations were qualitatively similar for males and females. An exception was sexual orientation: for both sexes, IWS was elevated for sexual minorities compared to heterosexual participants, but the difference was markedly bigger for males. In models adjusted for potential confounders and BMI at all ages, IWS was 0.09 S.D. higher (95% CI: −0.00, 0.18, p = 0.06) for sexual minority females, and 0.43 higher (95% CI: 0.29, 0.56, p < 0.001) for sexual minority males (interaction: 0.34 S.D., p < 0.001). Associations with IWS were also stronger among males than females for BMI at age 18 years (interaction: 0.11 S.D., p = 0.008), weight-based teasing at school (0.18 S.D., p = 0.04), family pressure to lose weight (interaction: 0.21 S. D·, p = 0.02), and stronger among females than males for having attended university (females: 0.09, S.D., 95% CI: 0.01, 0.18, p = 0.02, males: −0.06 S.D., 95% CI: −0.18, 0.05, p = 0.27, interaction: 0.14 S.D., p = 0.04). Using the 11-item version of the WBIS-M did not materially affect results (Tables S12–S15). Results of models using complete-case data were similar to results based on imputed data, but estimates less precise, owing to smaller sample sizes (Tables S16–S19).

## Discussion

Our study provides important evidence on the demographic, socioeconomic and family predictors of IWS. At age 31 years, IWS was elevated for females, sexual minorities, and socioeconomically disadvantaged adults, and this was not explained by differences in BMI in adulthood or earlier in life. Both family (as indexed by maternal qualifications) and adult (indexed by time spent not in education, employment, or training between age 21 and 29 years) socioeconomic history predicted IWS, independently of both child and adult BMI and confounders. People who had not attended university had higher IWS than those who had attended, but only among female participants was this independent of differences in adult BMI. Both male and female sexual minority participants had higher IWS than heterosexual counterparts, but this was stronger for males. In addition, several aspects of the social environment during development emerged as important for later IWS: negative weight-related comments from parents, weight-based teasing from family, and pressure to lose weight from family at age 13 years were robustly associated with IWS in adulthood, even after accounting for both child and adult BMI, underscoring the importance of the family environment. Peer relations were also important: as well as weight-based teasing at school, bullying for any reason was associated with IWS at age 31 years, especially in late adolescence and early adulthood. Lastly, feeling pressure from the media to lose weight at age 13 years was robustly associated with IWS at age 31 years, underscoring the importance of the wider social environment and its messaging about weight. Our results demonstrate that some of the higher IWS experienced by people with higher BMI in adulthood was explained by BMI during childhood and adolescence, further highlighting the importance of non-stigmatising messaging about weight for children and young people. Given substantial evidence that IWS has serious implications for mental and other aspect of health,^6^, ^7^, ^8^^,^^11^ these findings will be crucial for targeting prevention programmes and supporting people most at risk.

Higher IWS amongst female participants in our sample is consistent with most studies on IWS to date,^10^^,^^12^^,^^13^ and with evidence that women are more likely to experience weight-related discrimination.^9^ In contrast to US studies, which have reported lower IWS amongst African American participants, we did not observe any difference based on ethnicity. This suggests there are cultural differences between the US and Europe, consistent with evidence that weight stigma plays out in contextually specific ways.^23^ However, the small proportion of minority ethnic participants in ALSPAC meant power to detect such effects was low. Two socioeconomic indicators (maternal qualifications and NEET history) were independently associated with IWS, indicating that socioeconomic disadvantage in both early life and adulthood may shape personal experience of weight stigma. Results accord with a representative German study which found higher IWS among adults with lower income and fewer educational qualifications,^10^ and suggest that a person's early life socioeconomic circumstances may have long-lasting relevance, independent of educational attainment in adulthood. Findings also accord with evidence highlighting that stigmatizing attitudes about obesity and poverty, and particularly people receiving benefits, are closely connected.^28^ Our findings provide much needed evidence on sexual orientation predicting IWS, which is under-studied despite evidence that disordered eating is more prevalent among sexual minorities.^15^^,^^29^ Our results accord with earlier studies which find higher IWS among sexual minority men compared to heterosexual men,^15^, ^16^, ^17^ possibly reflecting idealization of a lean, muscular body type within gay culture.^15^^,^^16^ However, we also find higher IWS among sexual minority females than heterosexual females, which suggests other mechanisms. In addition to the factors driving IWS in all adults, stressors experienced by sexual minority women such as internalized homophobia or sexual orientation concealment may impact eating behaviour^29^ and in turn affect IWS. Relatedly, we identified bullying victimization in adolescence and adulthood as a risk factor for IWS, and experiencing bullying linked to sexual orientation may also contribute to IWS in this group. More broadly, research suggests that sexual minority women are not necessarily protected from beauty norms which value thinness, especially in the dating realm.^30^ Earlier studies based on psychology students^17^ or people engaged in weight management programmes^16^ found IWS to be similar for heterosexual and sexual minority women. The discrepancy may reflect cultural differences between the UK and US, or consequences of sample selection, and points to the need for research involving sexual minorities drawn from general population samples in diverse countries.

Our study accords with recent evidence on the importance of social processes in mediating links between body weight and psychological outcomes in adolescence.^31^ In particular, our findings highlight the importance of the family environment for later psychological outcomes; we found higher levels of IWS amongst those who had experienced negative weight-related comments, weight-based teasing, and pressure to lose weight from family, with these associations not explained by differences in adolescent and adult BMI. These results also align with studies suggesting that these factors may have long-term impacts on disordered eating.^19^^,^^20^ Findings regarding bullying in childhood and adolescence align with evidence linking these experiences with body image,^32^ while associations with bullying at age 23 years underscore both the continuation of bullying later in life, and its health consequences.^33^ Our results therefore indicate that bullying, which can be reduced following appropriate interventions,^34^ may be a fruitful target for intervention to reduce IWS and its consequences. Findings regarding perceived pressure from the media to lose weight align with substantial evidence of that weight stigma in the media is pervasive, and negatively affects attitudes about weight.^35^

This study examined IWS in a general population birth cohort study. By drawing on rich longitudinal data, we were able to investigate risk factors from childhood, adolescence, and early adulthood whilst minimising recall bias. Objective measurements of height and weight minimised measurement error in BMI, and availability of repeat measures through the life course meant associations with BMI at different stages of life could be robustly explored. However, since IWS was only included in the survey at age 31 years, influence of earlier IWS could not be explored. This may have affected reporting of exposures, such as weight-related comments at age 13 years, or influenced participants' NEET history, if people with high IWS are less likely to put themselves forwards for interviews or promotions. Although multiple imputation was used to reduce the impact of missing data in risk factors, we cannot be sure that data was missing at random, and for some variables the proportion of imputed data was relatively high. However, conclusions based on complete-case analysis did not differ, suggesting that our approach to handling missing data had not substantially impacted results. Relatedly, analysis was restricted to participants who completed the age 31 years questionnaire, and attrition by age 31 years may have influenced results. Our early adulthood BMI measure was from age 24 years, and some participants' weight may have changed between this and outcome measurement. However, tracking of BMI in this part of the life-course is likely to be high.^27^ Information was only available on participants’ biological sex as recorded at birth; consequently, how IWS relates to gender identity could not be explored using this data. We used a standard indicator of socioeconomic conditions in childhood (maternal qualifications), but IWS may relate differently to income-based measures, or educational qualifications of other family members. ALSPAC is a regional cohort study, and at baseline was not fully representative of the UK population, with single parent families, those living in rented accommodation and some ethnic minorities underrepresented. Replication is required in national studies, and in ethnically diverse European samples, given the low proportion of ethnic minority participants in ALSPAC. Future research in this and other surveys will be required to elucidate in more detail the mechanisms which link demographic, social and psychological risk factors across the life-course to IWS. As with any observational study, we have described associations; future work using other study designs will be required to establish if these associations are causal. Recent work has pointed to close links and possible conceptual overlap between IWS and body dissatisfaction and wider eating disorder cognitions,^36^ and future work should examine how these constructs are dynamically related over time. Meanwhile, further research on IWS among sexual minority women as well as men is needed to better understand IWS in this population.

### Conclusion

The current study has provided novel insights highlighting elevated IWS amongst females, sexual minorities, socioeconomically disadvantaged people, and those who in youth experience bullying, weight-related teasing, and feel under pressure to lose weight, especially from family members and the media. These differences are not explained by differences in BMI and point to strong and long-lasting effects on adult psychological health. These findings hold important implications for policy and practice relating to how we discuss weight in the media, in public spaces and families, and how we respond to bullying in schools, workplaces, and other settings. This is crucial considering how prevalent pressures to lose weight and weight-related stigma and discrimination are in many cultures around the world.

## Contributors

AMH: funding acquisition, conceptualization, formal analysis, visualization, writing–original draft, writing–review & editing. All other authors: supervision, writing–review & editing.

## Data sharing statement

Individual-level data from the ALSPAC birth cohort are not publicly available for reasons of clinical confidentiality. Data can be accessed after application to the ALSPAC Executive Team. Application instructions and data use agreements are available at http://www.bristol.ac.uk/alspac/researchers/access/.

## Declaration of interests

AMH, AAK, and ERR have no interests to declare. HB declares an NIHR Advanced Fellowship (NIHR302271) and support for attending meetings from the NIHR via the Bristol Biomedical Research Centre and is an elected member of the Faculty of Eating Disorders, RCPsych. LDH declares research grants unrelated to this work from the ESRC and British Heart Foundation. KC reports lecture fees from Ethicon and Apollo Endosurgery, consultation fees from Eli Lilly, Patient Advisory Board membership for Boehringer Ingelheim, and trusteeship for ASO. SWF declares researcher led grants from the National Institute for Health Research, the Office of Health Improvement and Disparities, Doncaster Council, the West Yorkshire Combined Authority, and Novo Nordisk; and support for attending academic conferences from Johnson & Johnson, Novo Nordisk, Devon NHS Integrated Care Service, the UK Parliament, and Safefood.

## References

[1] Baker C. (2023). https://researchbriefings.files.parliament.uk/documents/SN03336/SN03336.pdf.

[2] Emmer C., Bosnjak M., Mata J. (2020). The association between weight stigma and mental health: a meta-analysis. Obes Rev.

[3] Latner J.D., Barile J.P., Durso L.E., O'Brien K.S. (2014). Weight and health-related quality of life: the moderating role of weight discrimination and internalized weight bias. Eat Behav.

[4] Pearl R.L., Himmelstein M.S., Puhl R.M., Wadden T.A., Wojtanowski A.C., Foster G.D. (2019). Weight bias internalization in a commercial weight management sample: prevalence and correlates. Obes Sci Pract.

[5] Pearl R.L., Puhl R.M. (2014). Measuring internalized weight attitudes across body weight categories: validation of the modified weight bias internalization scale. Body Image.

[6] O'Brien K.S., Latner J.D., Puhl R.M. (2016). The relationship between weight stigma and eating behavior is explained by weight bias internalization and psychological distress. Appetite.

[7] Hilbert A., Braehler E., Haeuser W., Zenger M. (2014). Weight bias internalization, core self-evaluation, and health in overweight and obese persons. Obesity.

[8] Puhl R.M., Lessard L.M., Himmelstein M.S., Foster G.D. (2021). The roles of experienced and internalized weight stigma in healthcare experiences: perspectives of adults engaged in weight management across six countries. PLoS One.

[9] Spahlholz J., Baer N., König H.H., Riedel-Heller S.G., Luck-Sikorski C. (2016). Obesity and discrimination—a systematic review and meta-analysis of observational studies. Obes Rev.

[10] Hilbert A., Baldofski S., Zenger M., Löwe B., Kersting A., Braehler E. (2014). Weight bias internalization scale: psychometric properties and population norms. PLoS One.

[11] Marshall R.D., Latner J.D., Masuda A. (2020). Internalized weight bias and disordered eating: the mediating role of body image avoidance and drive for thinness. Front Psychol.

[12] Pearl R.L., Puhl R.M., Lessard L.M., Himmelstein M.S., Foster G.D. (2021). Prevalence and correlates of weight bias internalization in weight management: a multinational study. SSM Popul Health.

[13] Kliem S., Puls H.C., Hinz A., Kersting A., Brahler E., Hilbert A. (2020). Validation of a three-item short form of the modified weight bias internalization scale (WBIS-3) in the German population. Obes Facts.

[14] Puhl R.M., Himmelstein M.S., Quinn D.M. (2018). Internalizing weight stigma: prevalence and sociodemographic considerations in US adults. Obesity.

[15] Austen E., Greenaway K.H., Griffiths S. (2020). Differences in weight stigma between gay, bisexual, and heterosexual men. Body Image.

[16] Puhl R.M., Himmelstein M.S., Pearl R.L., Wojtanowski A.C., Foster G.D. (2019). Weight stigma among sexual minority adults: findings from a matched sample of adults engaged in weight management. Obesity.

[17] Shonrock A.T., Miller J.C., Byrd R. (2022). Experienced weight stigma, internalized weight bias, and maladaptive eating patterns among heterosexual and sexual minority individuals. Eat Weight Disord.

[18] Pearl R.L., Wadden T.A., Shaw Tronieri J. (2018). Sociocultural and familial factors associated with weight bias internalization. Obes Facts.

[19] Eisenberg M.E., Berge J.M., Fulkerson J.A., Neumark-Sztainer D. (2012). Associations between hurtful weight-related comments by family and significant other and the development of disordered eating behaviors in young adults. J Behav Med.

[20] Puhl R.M., Wall M.M., Chen C., Bryn Austin S., Eisenberg M.E., Neumark-Sztainer D. (2017). Experiences of weight teasing in adolescence and weight-related outcomes in adulthood: a 15-year longitudinal study. Prev Med.

[21] Pearl R.L., Puhl R.M. (2018). Weight bias internalization and health: a systematic review. Obes Rev.

[22] Munafò M.R., Tilling K., Taylor A.E., Evans D.M., Davey Smith G. (2018). Collider scope: when selection bias can substantially influence observed associations. Int J Epidemiol.

[23] Marini M., Sriram N., Schnabel K. (2013). Overweight people have low levels of implicit weight bias, but overweight nations have high levels of implicit weight bias. PLoS One.

[24] Northstone K., Lewcock M., Groom A. (2019). The Avon Longitudinal Study of Parents and Children (ALSPAC): an update on the enrolled sample of index children in 2019 [version 1; peer review: 2 approved]. Wellcome Open Res.

[25] Vidmar S.I., Cole T.J., Pan H. (2013). Standardizing anthropometric measures in children and adolescents with functions for Egen: update. Stata J.

[26] Wolke D., Woods S., Bloomfield L., Karstadt L. (2000). The association between direct and relational bullying and behaviour problems among primary school children. J Child Psychol Psychiatry.

[27] Kvaavik E., Tell G.S., Klepp K.I. (2003). Predictors and tracking of body mass index from adolescence into adulthood: follow-up of 18 to 20 Years in the Oslo youth study. Arch Pediatr Adolesc Med.

[28] Hughes A.M., McArthur D. (2023). Weight stigma, welfare stigma, and political values: evidence from a representative British survey. Soc Sci Med.

[29] Panza E., Fehling K.B., Pantalone D.W., Dodson S., Selby E.A. (2021). Multiply marginalized: linking minority stress due to sexual orientation, gender, and weight to dysregulated eating among sexual minority women of higher body weight. Psychol Sex Orientat Gend Divers.

[30] Gerend M.A., Stewart C., Wetzel K. (2022). Vulnerability and resilience to the harmful health consequences of weight discrimination in Black, Latina, and sexual minority women. Soc Sci Med.

[31] Blundell E., De Stavola B.L., Kellock M.D. (2024). Longitudinal pathways between childhood BMI, body dissatisfaction, and adolescent depression: an observational study using the UK Millennium Cohort Study. Lancet Psychiatr.

[32] Brixval C.S., Rayce S.L.B., Rasmussen M., Holstein B.E., Due P. (2012). Overweight, body image and bullying—an epidemiological study of 11- to 15-years olds. Eur J Public Health.

[33] Boudrias V., Trépanier S.G., Salin D. (2021). A systematic review of research on the longitudinal consequences of workplace bullying and the mechanisms involved. Aggress Violent Behav.

[34] Fraguas D., Díaz-Caneja C.M., Ayora M. (2021). Assessment of school anti-bullying interventions: a meta-analysis of randomized clinical trials. JAMA Pediatr.

[35] Kite J., Huang B.H., Laird Y. (2022). Influence and effects of weight stigmatisation in media: a systematic review. eClinicalMedicine.

[36] Romano K.A., Heron K.E., Sandoval C.M., Howard L.M., MacIntyre R.I., Mason T.B. (2022). A meta-analysis of associations between weight bias internalization and conceptually-related correlates: a step towards improving construct validity. Clin Psychol Rev.

