# Supplementary material for: Demographic, socioeconomic and life-course risk factors for internalized weight stigma in adulthood: evidence from an English birth cohort study
Source: Lancet Reg Health Eur. 2024 Apr 15;40:100895. doi: 10.1016/j.lanepe.2024.100895 (PMC11092882; doi:10.1016/j.lanepe.2024.100895)
Supplement: Supplementary Figure and Tables [file mmc1.docx]

**SUPPLEMENTARY MATERIAL: Socioeconomic and life-course risk factors for internalized weight stigma in adulthood: evidence from an English birth cohort study**

**Additional Methods**

The Modified Weight Bias Internalization Scale (WBIS-M)

Description of the ALSPAC cohort

Multiple imputation

Covariates included in regression models

Stata code showing model specification

**Figures**

Figure S1: Flow chart of inclusion and exclusion the sample

**Tables**

Table S1: Covariates included at each level of adjustment for all risk factors

Table S2: Comparison of retained and excluded participants based on complete-case data

Table S3: Percent of imputed data in analytic sample

*Main analyses*

Table S4: Demographic risk factors for internalized weight stigma at age 31

Table S5: BMI across development and internalized weight stigma at age 31

Table S6: Family, peer, and wider social influences at age 13 and internalized weight stigma at age 31

Table S7: Bullying victimization at age 8, 10, 12.5, 17.5 and 23 and internalized weight stigma at age 31

*Sex-stratified associations*

Table S8: Sex-stratified models: demographic risk factors for internalized weight stigma at age 31

Table S9: Sex-stratified models: BMI across development and internalized weight stigma at age 31

Table S10: Sex-stratified models: family, peer, and wider social environment at age 13 and internalized weight stigma at age 31

Table S11: Sex-stratified models: bullying victimization at age 8, 10, 12.5, 17.5 and 23 and internalized weight stigma at age 31

*11-item WBIS-M*

Table S12: Demographic risk factors for internalized weight stigma (11-item WBIS-M) at age 31

Table S13: BMI across development and internalized weight stigma (11-item WBIS-M) at age 31

Table S14: Family, peer, and wider social environment at age 13 and internalized weight stigma (11-item WBIS-M) at age 31

Table S15: Bullying victimization at age 8, 10, 12.5, 17.5 and 23 and internalized weight stigma (11-item WBIS-M) at age 31

Complete-case analysis

Table S16: Demographic risk factors for internalized weight stigma (complete-case analysis) at age 31

Table S17: BMI across development and internalized weight stigma (complete-case analysis) at age 31

Table S18: Family, peer, and wider social environment at age 13 and internalized weight stigma (complete-case analysis) at age 31

Table S19: Bullying victimization at age 8, 10, 12.5, 17.5 and 23 and internalized weight stigma (complete-case analysis) at age 31

**Additional Methods**

*The Modified Weight Bias Internalization Scale (WBIS-M)*

As part of a questionnaire sent out in 2022, all participants were asked to rate their agreement with the following statements, from 1 “does not apply to me at all” to 7 “applies to me perfectly”.

In the original 11-item WBIS-M^1^, the statement, “Because of my weight, I feel that I am just as competent as anyone” was included as item 1. Give recent studies supporting removal of this item from the original scale^2,3^, it was retained but moved to the end of the list.

1. I am less attractive than most other people because of my weight
2. I feel anxious about my weight because of what people might think of me
3. I wish I could drastically change my weight
4. Whenever I think a lot about my weight, I feel depressed
5. I hate myself because of my weight
6. My weight is a major way that I judge my value as a person
7. I don't feel that I deserve to have a really fulfilling social life, because of my weight
8. I am OK being the weight that I am*
9. Because of my weight, I don't feel like my true self
10. Because of my weight, I don't understand how anyone attractive would want to date me
11. Because of my weight, I feel that I am just as competent as anyone*

*reverse-coded.

In main analyses, a summary index was constructed by adding items 1-10 above. The 10 items showed very good internal consistency (Cronbach’s alpha=0.96). For the 0.9% of participants who had item-level missingness in one or two of these items, a prorated index value was calculated based on their completed responses. In a sensitivity analysis, an index considering all 11 items (Cronbach’s alpha=0.93) was used. Both indexes were standardized to have a mean of zero and standard deviation of one for analysis.

*Description of the ALSPAC cohort*

This analysis used data from the Avon Longitudinal Study of Parents and Children (ALSPAC), a pregnancy study of women with expected delivery dates between 1/4/1991 and 31/12/1992 who were living in or around Bristol ^4–6^. From the initial 14,541 pregnancies, 13,988 children were alive at 1 year. When the oldest children were around 7 years old, the study enrolled 913 additional children who had met the original eligibility criteria but had not been initially recruited. The total sample size for analyses using data collected after the age of seven is therefore 15,447 pregnancies, from which 14,901 children were alive at 1 year of age. The mothers, children, and mothers’ partners have been followed up through regular questionnaires and clinic assessments collecting data on biological, environmental, and lifestyle factors^5^. The initial sample was broadly representative of the UK population in the 1991 census, with underrepresentation of single parent families, those living in rented accommodation and some ethnic minorities^7^. Please note that the study website contains details of all the data that is available through a fully searchable data dictionary and variable search tool: <http://www.bristol.ac.uk/alspac/researchers/our-data/>. Ethical approval for the study was obtained from the ALSPAC Ethics and Law Committee and the Local Research Ethics Committee. Informed consent for the use of data collected via questionnaires and clinics was obtained from participants following the recommendations of the ALSPAC Ethics and Law Committee at the time. At age 18, study children were sent 'fair processing' materials describing ALSPAC’s intended use of their health and administrative records and given clear means to consent or object via a written form. Data were not extracted for participants who objected, or who were not sent fair processing materials. Data from 2014 onwards was collected using REDCap (Research Electronic Data Capture)^8^. REDCap is a secure, web-based software platform designed to support data capture for research studies hosted at the University of Bristol.

*Multiple imputation*.

Imputation models included the WBIS-M summary scale, all risk factors and covariates examined in analysis, and auxiliary variables to assist with imputation model convergence. For NEET history, separate binary variables for NEET status at each timepoint where this information was available were entered into imputation models, and a categorical variable derived post-imputation. Continuous variables were imputed using predictive mean matching, binary variables with logistic regression, and categorical variables with ordered or multinomial logistic regression. The % of each variable which was imputed is shown in Table S3.

*Covariates included in regression models*

Covariates included in every model are listed in Table S1. For each risk factor, a base model was run which adjusted for sex only. Subsequent models added additional factors which temporally preceded the risk factor and could potentially confound associations. For BMI at age 24, this included BMI at all previous timepoints. For family and wider social factors reported at age 13, we explored further adjustment for other family and wider social factors reported at the same time, and in bullying models, we explored further adjustment for BMI at the time and bullying at earlier and later timepoints. For bullying at age 23, the only BMI measurement close to the bullying report was shortly afterward (at age 24 years) and this was used instead. For all risk factors, a final model included BMI at age 24 years, to assess whether associations were independent of BMI in young adulthood.

*Stata code showing model specification.*

*Sex:

eststo kz021_m1: mi estimate, post: regress z_iws_10item i.kz021

eststo kz021_m2: mi estimate, post: regress z_iws_10item i.kz021 bmi_age24

eststo kz021_m3: mi estimate, post: regress z_iws_10item i.kz021 bmi_age24 f7ms102 fdms102 ff2034 fh3014 rescaled_bmi_age18

*Ethnicity:

eststo YP_ethnicity_composite_m1: mi estimate, post: regress z_iws_10item i.kz021 i.YP_ethnicity_composite

eststo YP_ethnicity_composite_m2: mi estimate, post: regress z_iws_10item i.kz021 i.YP_ethnicity_composite bmi_age24

eststo YP_ethnicity_composite_m3: mi estimate, post: regress z_iws_10item i.kz021 i.YP_ethnicity_composite bmi_age24 f7ms102 fdms102 ff2034 fh3014 rescaled_bmi_age18

*Mum's education:

eststo c_mum_edquals_3groups_R_m1: mi estimate, post: regress z_iws_10item i.kz021 i.c_mum_edquals_3groups_REV

eststo c_mum_edquals_3groups_R_m2: mi estimate, post: regress z_iws_10item i.kz021 i.c_mum_edquals_3groups_REV i.YP_ethnicity_composite

eststo c_mum_edquals_3groups_R_m3: mi estimate, post: regress z_iws_10item i.kz021 i.c_mum_edquals_3groups_REV i.YP_ethnicity_composite bmi_age24

eststo c_mum_edquals_3groups_R_m4: mi estimate, post: regress z_iws_10item i.kz021 i.c_mum_edquals_3groups_REV i.YP_ethnicity_composite bmi_age24 f7ms102 fdms102 ff2034 fh3014 rescaled_bmi_age18

*Sexual orientation:

eststo YP_sexorient_composite_m1: mi estimate, post: regress z_iws_10item i.kz021 i.YP_sexorient_composite

eststo YP_sexorient_composite_m2: mi estimate, post: regress z_iws_10item i.kz021 i.YP_sexorient_composite i.c_mum_edquals_3groups_REV i.YP_ethnicity_composite

eststo YP_sexorient_composite_m3: mi estimate, post: regress z_iws_10item i.kz021 i.YP_sexorient_composite i.c_mum_edquals_3groups_REV i.YP_ethnicity_composite bmi_age24

eststo YP_sexorient_composite_m4: mi estimate, post: regress z_iws_10item i.kz021 i.YP_sexorient_composite i.c_mum_edquals_3groups_REV i.YP_ethnicity_composite bmi_age24 f7ms102 fdms102 ff2034 fh3014 rescaled_bmi_age18

*Attended university:

eststo YPJ_attended_uni_REV_m1: mi estimate, post: regress z_iws_10item i.kz021 i.YPJ_attended_uni_REV

eststo YPJ_attended_uni_REV_m2: mi estimate, post: regress z_iws_10item i.kz021 i.YPJ_attended_uni_REV i.YP_ethnicity_composite i.c_mum_edquals_3groups_REV i.YP_sexorient_composite

eststo YPJ_attended_uni_REV_m3: mi estimate, post: regress z_iws_10item i.kz021 i.YPJ_attended_uni_REV i.YP_ethnicity_composite i.c_mum_edquals_3groups_REV i.YP_sexorient_composite bmi_age24

eststo YPJ_attended_uni_REV_m4: mi estimate, post: regress z_iws_10item i.kz021 i.YPJ_attended_uni_REV i.YP_ethnicity_composite i.c_mum_edquals_3groups_REV i.YP_sexorient_composite bmi_age24 f7ms102 fdms102 ff2034 fh3014 rescaled_bmi_age18

*NEET history:

eststo NEET_3group_m1: mi estimate, post: regress z_iws_10item i.kz021 i.NEET_3group

eststo NEET_3group_m2: mi estimate, post: regress z_iws_10item i.kz021 i.NEET_3group i.YP_ethnicity_composite i.c_mum_edquals_3groups_REV i.YP_sexorient_composite

eststo NEET_3group_m3: mi estimate, post: regress z_iws_10item i.kz021 i.NEET_3group i.YP_ethnicity_composite i.c_mum_edquals_3groups_REV i.YP_sexorient_composite bmi_age24

eststo NEET_3group_m4: mi estimate, post: regress z_iws_10item i.kz021 i.NEET_3group i.YP_ethnicity_composite i.c_mum_edquals_3groups_REV i.YP_sexorient_composite bmi_age24 f7ms102 fdms102 ff2034 fh3014 rescaled_bmi_age18

*BMI earlier in life:

foreach bmi in f7ms102 fdms102 ff2034 fh3014 rescaled_bmi_age18 {

*model 1: sex adjustment only

eststo `bmi'_m1: mi estimate, post: regress z_iws_10item i.kz021 `bmi'

*model 2: adjustment for all early-life demographic factors

eststo `bmi'_m2: mi estimate, post: regress z_iws_10item i.kz021 `bmi' i.YP_ethnicity_composite i.c_mum_edquals_3groups_REV i.YP_sexorient_composite

*model 3: adjustment for all demographic factors and bmi at 24

eststo `bmi'_m3: mi estimate, post: regress z_iws_10item i.kz021 `bmi' i.YP_ethnicity_composite i.c_mum_edquals_3groups_REV i.YP_sexorient_composite rescaled_bmi_age24

}

*BMI at 24:

foreach bmi in rescaled_bmi_age24 {

*model 1: sex adjustment only

eststo `bmi'_m1: mi estimate, post: regress z_iws_10item i.kz021 `bmi'

*model 2: adjustment for all early-life demographic factors

eststo `bmi'_m2: mi estimate, post: regress z_iws_10item i.kz021 `bmi' i.YP_ethnicity_composite i.c_mum_edquals_3groups_REV i.YP_sexorient_composite

*model 3: and other bmi measurements:

eststo `bmi'_m3: mi estimate, post: regress z_iws_10item i.kz021 `bmi' i.YP_ethnicity_composite i.c_mum_edquals_3groups_REV i.YP_sexorient_composite f7ms102 fdms102 ff2034 fh3014 rescaled_bmi_age18

*model 4: plus later demographic factors

eststo `bmi'_m4: mi estimate, post: regress z_iws_10item i.kz021 `bmi' i.YP_ethnicity_composite i.c_mum_edquals_3groups_REV i.YP_sexorient_composite f7ms102 fdms102 ff2034 fh3014 rescaled_bmi_age18 i.YPJ_attended_uni_REV i.NEET_3group

}

*Family, peer and wider social factors at age 13:

foreach var in md_comments family_teasing school_teasing press_family press_friends press_bfgf press_media {

*model 1: sex adjusted

eststo `var'_m1: mi estimate, post: regress z_iws_10item i.kz021 `var'

*model 2: adjusted for early life demographics:

eststo `var'_m2: mi estimate, post: regress z_iws_10item i.kz021 `var' i.YP_ethnicity_composite i.c_mum_edquals_3groups_REV YP_sexorient_composite

*model 3: with adjustment for age 12.5 BMI and other age 13 factors:

eststo `var'_m3: mi estimate, post: regress z_iws_10item i.kz021 `var' i.YP_ethnicity_composite i.c_mum_edquals_3groups_REV YP_sexorient_composite ff2034 md_comments family_teasing school_teasing press_family press_friends press_bfgf press_media

*model 4: with adjustment for age 12.5, other age 13 factors, and adult BMI:

eststo `var'_m4: mi estimate, post: regress z_iws_10item i.kz021 `var' i.YP_ethnicity_composite i.c_mum_edquals_3groups_REV i.YP_sexorient_composite ff2034 md_comments family_teasing school_teasing press_family press_friends press_bfgf press_media bmi_age24

}

*Bullying

foreach age in mc lc ea la ad {

*model 1: sex adjusted

eststo `age'_bullying_m1: mi estimate, post: regress z_iws_10item i.kz021 `age'_bullying

*model 2: demographic adjustment only

eststo `age'_bullying_m2: mi estimate, post: regress z_iws_10item i.kz021 `age'_bullying i.YP_ethnicity_composite i.c_mum_edquals_3groups_REV i.YP_sexorient_composite

*model 3: adjustment for all demographic factors, bmi at the time, and bullying at other timepoints

eststo `age'_bullying_m3: mi estimate, post: regress z_iws_10item i.kz021 `age'_bullying i.YP_ethnicity_composite i.c_mum_edquals_3groups_REV i.YP_sexorient_composite `age'_bmi mc_bullying lc_bullying ea_bullying la_bullying ad_bullying

*model 4: model 3 + BMI at age 24

eststo `age'_bullying_m4: mi estimate, post: regress z_iws_10item i.kz021 `age'_bullying i.YP_ethnicity_composite i.c_mum_edquals_3groups_REV i.YP_sexorient_composite `age'_bmi mc_bullying lc_bullying ea_bullying la_bullying ad_bullying ad_bmi

}

**Figure S1**: Flow chart of inclusion and exclusion the sample


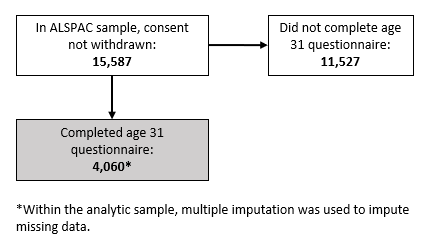


**Table S1: Covariates included at each level of adjustment for all risk factors**

| ***Demographic risk factors*** | | | | |
| --- | --- | --- | --- | --- |
|  | Model 1 | Model 2 | Model 3 |  |
| Sex | No covariates | BMI at age 24 |  |  |
| Ethnicity | Sex | Sex, BMI at age 24 | Sex, BMI at age 7, 10, 12.5, 15.5, 17.5, and 24 |  |
| Mother’s educational qualifications | Sex | Sex, ethnicity | Sex, ethnicity, BMI at age 24 | Sex, ethnicity, BMI at age 7, 10, 12.5, 15.5, 17.5, and 24 |
| Sexual orientation | Sex | Sex, ethnicity, mother’s educational qualifications | Sex, ethnicity, mother’s educational qualifications, BMI at age 24 | Sex, ethnicity, mother’s educational qualifications, BMI at age 7, 10, 12.5, 15.5, 17.5, and 24 |
| Attended university by age 30 | Sex | Sex, ethnicity, mother’s educational qualifications, sexual orientation | Sex, ethnicity, mother’s educational qualifications, sexual orientation, BMI at age 24 | Sex, ethnicity, mother’s educational qualifications, sexual orientation, BMI at age 7, 10, 12.5, 15.5, 17.5, and 24 |
| Occasions not in education, employment, or training (NEET) between age 21 and age 29 years | Sex | Sex, ethnicity, mother’s educational qualifications, sexual orientation | Sex, ethnicity, mother’s educational qualifications, sexual orientation,  BMI at age 24 | Sex, ethnicity, mother’s educational qualifications, sexual orientation,  BMI at age 7, 10, 12.5, 15.5, 17.5, and 24 |
| ***BMI from age 7 years to age 24 years*** | | | | |
|  | Model 1 | Model 2 | Model 3 | Model 4 |
| BMI at age 7, 10, 12.5, 15.5, and 17.5 | Sex | Sex, ethnicity, mother’s educational qualifications, sexual orientation | Sex, ethnicity, mother’s educational qualifications, sexual orientation, BMI at age 24 years |  |
| BMI at age 24 | Sex | Sex, ethnicity, mother’s educational qualifications, sexual orientation | Sex, ethnicity, mother’s educational qualifications, sexual orientation, BMI at ages 7, 10, 12.5, 15.5, and 17.5 years | Sex, ethnicity, mother’s educational qualifications, sexual orientation, BMI at ages 7, 10, 12.5, 15.5, and 17.5 years, university attendance and NEET history |
| ***Family, peer, and wider social environment at age 13 years*** | | | | |
|  | Model 1 | Model 2 | Model 3 | Model 4 |
| All age 13 factors^a^ | Sex | Sex, ethnicity, mother’s educational qualifications, sexual orientation | Sex, ethnicity, mother’s educational qualifications, sexual orientation, BMI at age 12.5 years, other age 13 factors | Sex, ethnicity, mother’s educational qualifications, sexual orientation, BMI at age 12.5 years, other age 13 factors, BMI at age 24 years |
|  | Model 1 | Model 2 | Model 3 | Model 4 |
| Bullying victimization at ages 8, 10, 12.5, 17.5, and 23 years | Sex | Sex, ethnicity, mother’s educational qualifications, sexual orientation | Sex, ethnicity, mother’s educational qualifications, sexual orientation, BMI at the time^b^, bullying at all other timepoints | Sex, ethnicity, mother’s educational qualifications, sexual orientation, BMI at the time, bullying at all other timepoints, BMI at age 24 years |
| ^a^The following were reported at age 13: parents make negative comments about young person’s weight; teased by family about weight/shape; teased at school about weight/shape; pressure to lose weight from friends; pressure to lose weight from family; pressure to lose weight from girls/ boys the young person has gone out with; pressure to lose weight from media.  ^b^Models included BMI at age 8 (bullying at age 8), BMI at age 10 (bullying at age 10) BMI at 12.5 (bullying at 12.5), BMI at 17.5 (bullying at 17.5) and BMI at 24 (bullying at 23). | | | | |

**Table S2: Descriptive characteristics of excluded and retained participants, based on complete-case data^a^**

| **Continuous variables** |  | **Excluded** |  | **Retained** |  | **P (t-test/ chi2)** |
| --- | --- | --- | --- | --- | --- | --- |
|  |  | ***Mean (SD)*** | ***N*** | ***Mean (SD)*** | ***N*** |  |
| Modified Internalized Weight Bias Score (WBIS-M)^b^ |  | - | 0 | 20.1 (17.0) | 4,038 | **-** |
| BMI z-score at age 7 |  | 0.2 (1.1) | 4,993 | - 1. (1.0) | 3,186 | **0.01** |
| BMI z-score at age 10 |  | 0.4 (1.2) | 4,300 | 0.3 (1.2) | 3,145 | **0.005** |
| BMI z-score at age 12.5 |  | 0.4 (1.2) | 3,695 | 0.3 (1.2) | 2,988 | **0.02** |
| BMI z-score at age 15.5 |  | 0.4 (1.1) | 2,713 | 0.3 (1.1) | 2,687 | **0.02** |
| BMI (kg/m^2^) at age 18 |  | 23.1 (4.3) | 2,368 | 22.7 (4.2) | 2,684 | **0.003** |
| BMI (kg/m^2^) at age 24 |  | 25.2 (5.0) | 1,267 | 24.8 (5.1) | 2,704 | **0.04** |
| **Categorical variables** | **category** | ***%*** | ***N*** | ***%*** | ***N*** |  |
| Sex | Female | 42.4 | 10,773 | 66.4 | 4,060 | **<0.001** |
|  | Male | 57.6 |  | 33.6 |  |  |
| Ethnicity | White | 94.4 | 8,564 | 95.9 | 3,962 | **<0.001** |
|  | Any other ethnicity | 5.6 |  | 4.1 |  |  |
| Mother’s educational qualifications | GCSEs/O-levels/no qualifications | 58.2 | 8,732 | 46.7 | 3,633 | **<0.001** |
|  | A-levels/vocational qualification | 31.5 |  | 34.3 |  |  |
|  | University degree | 10.3 |  | 19.1 |  |  |
| Attended university by age 30 | No | 33.5 | 1,701 | 32.8 | 3,821 | **0.60** |
|  | Yes | 66.5 |  | 67.2 |  |  |
| Times not in education, employment, or training (NEET) between age 21 and age 31 | Never | 81.8 | 99 | 79.9 | 1,489 | **0.80** |
|  | Once | 13.1 |  | 15.6 |  |  |
|  | Twice or more | 5.1 |  | 4.6 |  |  |
| Sexual orientation | Heterosexual | 79.3 | 1,239 | 85.8 | 3,953 | **<0.001** |
|  | Any other orientation | 20.7 |  | 14.2 |  |  |
| Age 13: parents make negative comments about young person’s weight | Yes | 76.3 | 3,098 | 73.9 | 2,978 | **0.03** |
|  | No | 23.7 |  | 26.1 |  |  |
| Age 13: teased by family about weight/shape | Yes | 78.0 | 3,107 | 76.7 | 2,995 | **0.21** |
|  | No | 22.0 |  | 23.3 |  |  |
| Age 13: teased at school about weight/shape | Yes | 77.3 | 3,107 | 76.9 | 2,987 | **0.70** |
|  | No | 22.7 |  | 23.1 |  |  |
| Age 13: pressure to lose weight from friends | Yes | 80.1 | 3,107 | 77.5 | 2,993 | **0.001** |
|  | No | 19.1 |  | 22.6 |  |  |
| Age 13: pressure to lose weight from family | Yes | 80.5 | 3,104 | 77.7 | 2,991 | **0.006** |
|  | No | 19.5 |  | 22.3 |  |  |
| Age 13: pressure to lose weight from girls/boys the young person has gone out with | Yes | 87.9 | 3,100 | 87.3 | 2,998 | **0.49** |
|  | No | 12.1 |  | 12.7 |  |  |
| Age 13: pressure to lose weight from media | Yes | 72.4 | 3,104 | 65.8 | 2,987 | **<0.001** |
|  | No | 27.6 |  | 34.2 |  |  |
| Bullying victimization: age 8 clinic | Yes | 56.5 | 4,110 | 63.8 | 2,897 | **<0.001** |
|  | No | 43.5 |  | 36.2 |  |  |
| Bullying victimization: age 10 clinic | Yes | 72.4 | 4,174 | 78.3 | 3,068 | **<0.001** |
|  | No | 27.6 |  | 21.7 |  |  |
| Bullying victimization: age 12.5 clinic | Yes | 44.9 | 3,705 | 45.8 | 3,026 | **0.46** |
|  | No | 55.1 |  | 54.2 |  |  |
| Bullying victimization: age 17.5 clinic | Yes | 73.8 | 1,811 | 71.3 | 2,247 | **0.07** |
|  | No | 26.2 |  | 28.8 |  |  |
| Bullying victimization: age 23 questionnaire | Yes | 79.9 | 1,207 | 82.2 | 2,858 | **0.08** |
|  | No | 20.1 |  | 17.8 |  |  |
| ^a^The analytic sample was restricted to 4,060 participants who completed the age 31 questionnaire. ^b^Possible range 0-60. | | | | | | |

**Table S3: Percent of imputed data in analytic sample**

| Variable | ***% imputed*** |
| --- | --- |
| Modified Internalized Weight Bias Score (WBIS-M) | 0.5 |
| BMI z-score at age 7 | 21.5 |
| BMI z-score at age 10 | 22.5 |
| BMI z-score at age 12.5 | 26.4 |
| BMI z-score at age 15.5 | 33.8 |
| BMI (kg/m^2^) at age 17.5 | 33.9 |
| BMI (kg/m^2^) at age 24 | 33.4 |
| Sex | 0.0 |
| Ethnicity | 2.4 |
| Mother’s educational qualifications | 10.5 |
| Attended university by age 30^a^ | 5.9 |
| Times not in education, employment, or training (NEET) between age 21 and age 31^b^ | 63.3 |
| Sexual orientation | 2.6 |
| Age 13: parents make negative comments about young person’s weight | 26.7 |
| Age 13: teased by family about weight/shape | 26.2 |
| Age 13: teased at school about weight/shape | 26.4 |
| Age 13: pressure to lose weight from friends | 26.3 |
| Age 13: pressure to lose weight from family | 26.3 |
| Age 13: pressure to lose weight from girls/boys the young person has gone out with | 26.2 |
| Age 13: pressure to lose weight from media | 26.4 |
| Bullying victimization: age 8 clinic | 28.6 |
| Bullying victimization: age 10 clinic | 24.4 |
| Bullying victimization: age 12.5 clinic | 25.4 |
| Bullying victimization: age 17.5 clinic | 44.7 |
| Bullying victimization: age 23 questionnaire | 29.6 |
| ^a^At age 26 and age 30, participants reported whether they had attended, or were currently attending, university. Participants missing both reports were assigned a ‘yes’ if in at age 22, 23, 24 or 25 they had reported graduating from university in the past 12 months. Remaining missing values were imputed. ^b^This was constructed post-imputation by combining information from variables on employment status at 6 timepoints (age 21, 22, 23, 25, 27, and 29). 63.3% of participants had missingness in at least one of these. | |

|  | **Model1^a^** | | | | **Model2** | | | | **Model 3** | | | | **Model 4** | | | |
| --- | --- | --- | --- | --- | --- | --- | --- | --- | --- | --- | --- | --- | --- | --- | --- | --- |
|  | Beta | LCI | UCI | LCI | LCI | LCI | LCI | p | Beta | LCI | UCI | p | Beta | LCI | UCI | p |
| Male | Ref |  |  |  | Ref |  |  |  | Ref |  |  |  |  |  |  |  |
| Female | 0.57 | 0.51 | 0.64 | <0.001 | 0.54 | 0.49 | 0.60 | <0.001 | 0.56 | 0.50 | 0.61 | <0.001 |  |  |  |  |
| White | Ref |  |  |  | Ref |  |  |  |  |  |  |  |  |  |  |  |
| Any other ethnicity | 0.00 | -0.16 | 0.15 | 0.99 | 0.00 | -0.14 | 0.14 | 0.99 | 0.00 | -0.14 | 0.13 | 0.97 |  |  |  |  |
| Maternal qualifications: University degree | Ref |  |  |  | Ref |  |  |  | Ref |  |  |  |  |  |  |  |
| A-levels/vocational qualifications | 0.19 | 0.10 | 0.28 | <0.001 | 0.19 | 0.10 | 0.28 | <0.001 | 0.10 | 0.02 | 0.18 | 0.01 | 0.10 | 0.02 | 0.18 | 0.01 |
| GCSEs/O-levels or less | 0.31 | 0.23 | 0.40 | <0.001 | 0.31 | 0.23 | 0.40 | <0.001 | 0.17 | 0.09 | 0.25 | <0.001 | 0.16 | 0.08 | 0.24 | <0.001 |
| Sexual orientation: heterosexual | Ref |  |  |  | Ref |  |  |  | Ref |  |  |  |  |  |  |  |
| Sexual orientation: any other orientation | 0.28 | 0.19 | 0.36 | <0.001 | 0.29 | 0.21 | 0.37 | <0.001 | 0.17 | 0.10 | 0.25 | <0.001 | 0.17 | 0.09 | 0.24 | <0.001 |
| University attendance by age 30: yes |  |  |  |  | Ref |  |  |  | Ref |  |  |  |  |  |  |  |
| University attendance by age 30: no | 0.24 | 0.18 | 0.31 | <0.001 | 0.20 | 0.13 | 0.26 | <0.001 | 0.05 | -0.02 | 0.11 | 0.16 | 0.04 | -0.02 | 0.10 | 0.18 |
| NEET age 21-31: never | Ref |  |  |  | Ref |  |  |  | Ref |  |  |  |  |  |  |  |
| NEET age 21-31: once | 0.08 | -0.01 | 0.18 | 0.09 | 0.08 | -0.02 | 0.17 | 0.123 | 0.04 | -0.04 | 0.13 | 0.32 | 0.05 | -0.04 | 0.13 | 0.29 |
| NEET age 21-31: 2+ times | 0.34 | 0.21 | 0.47 | <0.001 | 0.30 | 0.17 | 0.42 | <0.001 | 0.18 | 0.06 | 0.30 | 0.004 | 0.20 | 0.08 | 0.32 | 0.001 |
| ^a^Model 1 adjusts for no covariates (sex model) or sex only (all other models). Model 2 adjusts for BMI at age 24 (sex model, ethnicity model), sex and ethnicity (maternal qualifications model), sex, ethnicity, and maternal qualifications (sexual orientation), or sex, ethnicity, maternal qualifications, and sexual orientation (university attendance and NEET history). Model 3: model 2 + BMI at age 24 (all except sex and ethnicity models) or model 2 + BMI at ages 7, 10, 12.5, 15.5, 17.5 years. Model 4: Model 3 + BMI at ages 7, 10, 12.5, 15.5, 17.5 years. | | | | | | | | | | | | | | | | |

**Table S4: Demographic risk factors for internalized weight stigma at age 31**

| ***BMI at age 7 to 17.5 and internalized weight stigma at age 31^a^*** | | | | | | | | | | | | | | | | |
| --- | --- | --- | --- | --- | --- | --- | --- | --- | --- | --- | --- | --- | --- | --- | --- | --- |
|  | **Model1** | | | | **Model2** | | | | **Model3** | | | |  |  |  |  |
|  | Beta | LCI | UCI | p | Beta | LCI | UCI | p | Beta | LCI | UCI | p |  |  |  |  |
| BMI z-score at 7 | 0.28 | 0.25 | 0.31 | <0.001 | 0.27 | 0.24 | 0.3 | <0.001 | 0.06 | 0.02 | 0.09 | 0.001 |  |  |  |  |
| BMI z-score at 10 | 0.3 | 0.28 | 0.33 | <0.001 | 0.29 | 0.27 | 0.32 | <0.001 | 0.10 | 0.07 | 0.13 | <0.001 |  |  |  |  |
| BMI z-score at 12.5 | 0.32 | 0.30 | 0.35 | <0.001 | 0.31 | 0.29 | 0.34 | <0.001 | 0.12 | 0.09 | 0.15 | <0.001 |  |  |  |  |
| BMI z-score at 15.5 | 0.34 | 0.32 | 0.37 | <0.001 | 0.33 | 0.30 | 0.36 | <0.001 | 0.10 | 0.06 | 0.14 | <0.001 |  |  |  |  |
| BMI at 17.5 (per 5kg/m^2^) | 0.46 | 0.43 | 0.50 | <0.001 | 0.45 | 0.41 | 0.48 | <0.001 | 0.11 | 0.04 | 0.17 | 0.001 |  |  |  |  |
| ***^a^***Model 1 adjusts for sex. Model 2: Model 1 + ethnicity, maternal qualifications, and sexual orientation. Model 3: Model 2 + BMI at age 24. | | | | | | | | | | | | | | | | |
| ***BMI at age 24 and internalized weight stigma at age 31^b^*** | | | | | | | | | | | | | | | | |
|  | **Model1** | | | | **Model2** | | | | **Model3** | | | | **Model4** | | | |
|  | Beta | LCI | UCI | p | Beta | LCI | UCI | p | Beta | LCI | UCI | p | Beta | LCI | UCI | p |
| BMI at 24 (per 5kg/m^2^) | 0.44 | 0.41 | 0.47 | <0.001 | 0.43 | 0.4 | 0.45 | <0.001 | 0.33 | 0.28 | 0.38 | <0.001 | 0.32 | 0.27 | 0.37 | <0.001 |
| ***^b^***Model 1 adjusts for sex. Model 2: Model 1 + ethnicity, maternal qualifications, and sexual orientation. Model 3: Model 2 + BMI at ages 7, 10, 12.5, 15.5, and 17.5. Model 4: Model 3 + university attendance and NEET history. | | | | | | | | | | | | | | | | |

**Table S5: BMI across development and internalized weight stigma at age 31**

|  | **Model1^a^** | | | | **Model2** | | | | **Model3** | | | | **Model4** | | | |
| --- | --- | --- | --- | --- | --- | --- | --- | --- | --- | --- | --- | --- | --- | --- | --- | --- |
|  | Beta | LCI | UCI | p | Beta | LCI | UCI | p | Beta | LCI | UCI | p | Beta | LCI | UCI | p |
| Negative weight-based comments from parents | 0.56 | 0.49 | 0.64 | <0.001 | 0.55 | 0.47 | 0.62 | <0.001 | 0.17 | 0.09 | 0.26 | <0.001 | 0.14 | 0.05 | 0.22 | 0.002 |
| Weight-based teasing: family | 0.43 | 0.35 | 0.52 | <0.001 | 0.42 | 0.34 | 0.50 | <0.001 | 0.13 | 0.04 | 0.22 | 0.003 | 0.14 | 0.05 | 0.22 | 0.002 |
| Weight-based teasing at school | 0.47 | 0.39 | 0.55 | <0.001 | 0.45 | 0.37 | 0.52 | <0.001 | 0.18 | 0.10 | 0.26 | <0.001 | 0.09 | 0.02 | 0.17 | 0.02 |
| Pressure to lose weight: family | 0.65 | 0.57 | 0.73 | <0.001 | 0.63 | 0.55 | 0.71 | <0.001 | 0.14 | 0.03 | 0.24 | 0.009 | 0.13 | 0.03 | 0.23 | 0.01 |
| Pressure to lose weight: friends | 0.48 | 0.41 | 0.56 | <0.001 | 0.47 | 0.39 | 0.55 | <0.001 | 0.04 | -0.05 | 0.12 | 0.44 | 0.05 | -0.04 | 0.13 | 0.26 |
| Pressure to lose weight: boys/girls | 0.41 | 0.30 | 0.51 | <0.001 | 0.39 | 0.29 | 0.49 | <0.001 | -0.03 | -0.14 | 0.07 | 0.52 | -0.01 | -0.11 | 0.10 | 0.92 |
| Pressure to lose weight: media | 0.52 | 0.45 | 0.59 | <0.001 | 0.51 | 0.44 | 0.58 | <0.001 | 0.17 | 0.09 | 0.25 | <0.001 | 0.17 | 0.10 | 0.25 | <0.001 |
| **^a^** Model 1 adjusts for sex. Model 2: Model 1 + ethnicity, maternal qualifications, and sexual orientation. Model 3: Model 2 + BMI at age 12.5 years and other age 13 factors. Model 4: Model 3 + BMI at age 24 years. | | | | | | | | | | | | | | | | |

**Table S6: Family, peer, and wider social environment at age 13 years and internalized weight stigma at age 31***

|  | **Model1** | | | | **Model2** | | | | **Model3** | | | | **Model4** | | | |
| --- | --- | --- | --- | --- | --- | --- | --- | --- | --- | --- | --- | --- | --- | --- | --- | --- |
| ***Any bullying*** | Beta | LCI | UCI | p | Beta | LCI | UCI | p | Beta | LCI | UCI | p | Beta | LCI | UCI | p |
| Age 8 | 0.14 | 0.07 | 0.22 | <0.001 | 0.13 | 0.05 | 0.20 | 0.001 | 0.05 | -0.03 | 0.12 | 0.21 | 0.02 | -0.04 | 0.09 | 0.47 |
| Age 10 | 0.20 | 0.11 | 0.28 | <0.001 | 0.18 | 0.10 | 0.26 | <0.001 | 0.09 | 0.01 | 0.17 | 0.04 | 0.06 | -0.02 | 0.13 | 0.14 |
| Age 12.5 | 0.13 | 0.06 | 0.20 | 0.001 | 0.11 | 0.04 | 0.18 | 0.002 | 0.02 | -0.05 | 0.08 | 0.65 | 0.04 | -0.03 | 0.10 | 0.24 |
| Age 17.5 | 0.31 | 0.22 | 0.39 | <0.001 | 0.28 | 0.20 | 0.37 | <0.001 | 0.21 | 0.13 | 0.28 | <0.001 | 0.19 | 0.11 | 0.26 | <0.001 |
| Age 23 | 0.41 | 0.32 | 0.50 | <0.001 | 0.37 | 0.28 | 0.46 | <0.001 | 0.25 | 0.17 | 0.33 | <0.001 | 0.25 | 0.17 | 0.33 | <0.001 |
|  | **Model1** | | | | **Model2** | | | | **Model3** | | | | **Model4** | | | |
| ***Bullying types*** | Beta | LCI | UCI | p | Beta | LCI | UCI | p | Beta | LCI | UCI | p | Beta | LCI | UCI | p |
| Age 8 - direct | 0.13 | 0.05 | 0.21 | 0.002 | 0.12 | 0.04 | 0.21 | 0.003 | 0.05 | -0.04 | 0.13 | 0.29 | 0.01 | -0.06 | 0.09 | 0.70 |
| Age 8 - relational | 0.07 | -0.05 | 0.19 | 0.23 | 0.04 | -0.07 | 0.16 | 0.45 | 0.01 | -0.09 | 0.12 | 0.82 | 0.03 | -0.06 | 0.13 | 0.48 |
| Age 10 - direct | 0.13 | 0.03 | 0.22 | 0.01 | 0.11 | 0.01 | 0.21 | 0.02 | 0.04 | -0.05 | 0.13 | 0.38 | 0.03 | -0.06 | 0.11 | 0.56 |
| Age 10 - relational | 0.20 | 0.05 | 0.35 | 0.008 | 0.19 | 0.05 | 0.34 | 0.01 | 0.09 | -0.04 | 0.22 | 0.19 | 0.07 | -0.05 | 0.19 | 0.26 |
| Age 12.5 - direct | 0.08 | 0.00 | 0.15 | 0.06 | 0.06 | -0.02 | 0.13 | 0.15 | -0.04 | -0.11 | 0.03 | 0.22 | -0.01 | -0.08 | 0.05 | 0.66 |
| Age 12.5 - relational | 0.12 | 0.03 | 0.21 | 0.007 | 0.12 | 0.04 | 0.21 | 0.005 | 0.08 | 0.00 | 0.16 | 0.04 | 0.07 | 0.00 | 0.15 | 0.06 |
| Age 17.5 - direct | 0.31 | 0.18 | 0.44 | <0.001 | 0.28 | 0.15 | 0.41 | <0.001 | 0.11 | -0.01 | 0.24 | 0.07 | 0.08 | -0.04 | 0.19 | 0.20 |
| Age 17.5 – relational | 0.18 | 0.08 | 0.28 | <0.001 | 0.16 | 0.06 | 0.26 | 0.001 | 0.16 | 0.07 | 0.24 | 0.001 | 0.16 | 0.07 | 0.24 | <0.001 |
| Age 17.5 - cyber | 0.14 | -0.03 | 0.30 | 0.10 | 0.14 | -0.02 | 0.31 | 0.08 | 0.13 | -0.02 | 0.27 | 0.08 | 0.10 | -0.04 | 0.24 | 0.17 |
| Age 23 - direct | 0.37 | 0.20 | 0.54 | <0.001 | 0.34 | 0.17 | 0.51 | <0.001 | 0.28 | 0.13 | 0.44 | <0.001 | 0.28 | 0.13 | 0.44 | <0.001 |
| Age 23 – relational | 0.31 | 0.20 | 0.42 | <0.001 | 0.28 | 0.17 | 0.39 | <0.001 | 0.16 | 0.06 | 0.25 | 0.001 | 0.16 | 0.06 | 0.25 | 0.001 |
| Age 23 - cyber | 0.11 | -0.10 | 0.33 | 0.29 | 0.10 | -0.11 | 0.31 | 0.36 | 0.12 | -0.09 | 0.32 | 0.26 | 0.12 | -0.09 | 0.32 | 0.26 |
| **^a^**Model 1 adjusts for sex. Model 2 adjusts for sex, ethnicity, maternal qualifications, and sexual orientation. Model 3: model 2 + BMI at the time and bullying at other timepoints. Model 4: Model 3 + BMI at age 24 years. | | | | | | | | | | | | | | | | |

**Table S7: Bullying victimization at age 8, 10, 12.5, 17.5 and 23 and internalized weight stigma at age 31^a^**

**Table S8: Sex stratified models: demographic risk factors for internalized weight stigma at age 31**^a^

|  | **Model1**^a^ | | | | **Model2** | | | | **Model3** | | | | **Model 4** | | | |  |
| --- | --- | --- | --- | --- | --- | --- | --- | --- | --- | --- | --- | --- | --- | --- | --- | --- | --- |
| **Females** | Beta | LCI | UCI | p | Beta | LCI | UCI | p | Beta | LCI | UCI | p | Beta | LCI | UCI | p |  |
| White | Ref |  |  |  | Ref |  |  |  | Ref |  |  |  |  |  |  |  |  |
| Any other ethnicity | -0.03 | -0.23 | 0.16 | 0.74 | -0.03 | -0.21 | 0.15 | 0.75 | 0.00 | -0.18 | 0.17 | 0.98 |  |  |  |  |  |
| Maternal qualifications: university degree | Ref |  |  |  | Ref |  |  |  | Ref |  |  |  |  |  |  |  |  |
| A-levels/vocational qualifications | 0.24 | 0.12 | 0.35 | <0.001 | 0.24 | 0.12 | 0.35 | <0.001 | 0.15 | 0.04 | 0.25 | 0.006 | 0.15 | 0.04 | 0.25 | 0.005 |  |
| GCSEs/O-levels or less | 0.35 | 0.24 | 0.46 | <0.001 | 0.35 | 0.24 | 0.46 | <0.001 | 0.19 | 0.09 | 0.29 | <0.001 | 0.18 | 0.08 | 0.28 | <0.001 |  |
| Sexual orientation: heterosexual | Ref |  |  |  | Ref |  |  |  | Ref |  |  |  |  |  |  |  |  |
| Sexual orientation: any other orientation | 0.19 | 0.08 | 0.29 | <0.001 | 0.21 | 0.11 | 0.32 | <0.001 | 0.09 | 0.00 | 0.19 | 0.05 | 0.09 | -0.00 | 0.18 | 0.06 |  |
| University attendance by age 30: yes | Ref |  |  |  | Ref |  |  |  | Ref |  |  |  |  |  |  |  |  |
| University attendance by age 30: no | 0.31 | 0.23 | 0.39 | <0.001 | 0.26 | 0.18 | 0.35 | <0.001 | 0.09 | 0.01 | 0.17 | 0.02 | 0.09 | 0.01 | 0.17 | 0.02 |  |
|  | **Model1** | | | | **Model2** | | | | **Model3** | | | |  |  |  |  |  |
| **Males** | Beta | LCI | UCI | p | Beta | LCI | UCI | p | Beta | LCI | UCI | p |  |  |  |  | p (sex interaction)  in fully-adjusted model |
| White | Ref |  |  |  | Ref |  |  |  | Ref |  |  |  |  |  |  |  |  |
| Any other ethnicity | 0.06 | -0.21 | 0.32 | 0.69 | 0.05 | -0.21 | 0.30 | 0.72 | -0.01 | -0.26 | 0.24 | 0.93 |  |  |  |  | 0.93 |
| Maternal qualifications: university degree | Ref |  |  |  | Ref |  |  |  |  |  |  |  |  |  |  |  |  |
| A-levels/vocational qualifications | 0.09 | -0.06 | 0.24 | 0.22 | 0.09 | -0.06 | 0.24 | 0.22 | 0.02 | -0.12 | 0.16 | 0.77 | 0.01 | -0.13 | 0.15 | 0.88 | 0.13 |
| GCSEs/O-levels or less | 0.27 | 0.12 | 0.42 | <0.001 | 0.27 | 0.12 | 0.42 | <0.001 | 0.18 | 0.04 | 0.32 | 0.01 | 0.18 | 0.04 | 0.32 | 0.01 | 0.99 |
| Sexual orientation: heterosexual | Ref |  |  |  | Ref |  |  |  | Ref | Ref |  |  |  |  |  |  |  |
| Sexual orientation: any other orientation | 0.56 | 0.42 | 0.71 | <0.001 | 0.56 | 0.42 | 0.71 | <0.001 | 0.46 | 0.32 | 0.59 | <0.001 | 0.43 | 0.29 | 0.56 | <0.001 | <0.001 |
| University attendance by age 30: yes | Ref |  |  |  | Ref |  |  |  | Ref | Ref |  |  |  |  |  |  |  |
| University attendance by age 30: no | 0.09 | -0.03 | 0.21 | 0.13 | 0.03 | -0.09 | 0.15 | 0.62 | -0.05 | -0.17 | 0.07 | 0.40 | -0.06 | -0.18 | 0.05 | 0.27 | 0.04 |
| ^a^Model 1 adjusts for no covariates. Model 2 adjusts for BMI at age 24 (ethnicity model), ethnicity (maternal qualifications model), ethnicity and maternal qualifications (sexual orientation), or ethnicity, maternal qualifications, and sexual orientation (university attendance). Model 3: model 2 + BMI at age 24 (all except ethnicity model), or model 2 + BMI at ages 7, 10, 12.5, 15.5, 17.5 years (ethnicity model). Model 4: Model 3 + BMI at ages 7, 10, 12.5, 15.5, 17.5 years. | | | | | | | | | | | | | | | | | |

|  | **Model1** | | | | **Model2** | | | | **Model3** | | | |  | | | |  |
| --- | --- | --- | --- | --- | --- | --- | --- | --- | --- | --- | --- | --- | --- | --- | --- | --- | --- |
| **Females** | Beta | LCI | UCI | p | Beta | LCI | UCI | p | Beta | LCI | UCI | p |  |  |  |  |  |
| BMI z-score at 7^a^ | 0.31 | 0.28 | 0.35 | <0.001 | 0.31 | 0.27 | 0.35 | <0.001 | 0.09 | 0.04 | 0.13 | <0.001 |  |  |  |  |  |
| BMI z-score at 10^a^ | 0.34 | 0.30 | 0.37 | <0.001 | 0.33 | 0.29 | 0.36 | <0.001 | 0.13 | 0.09 | 0.17 | <0.001 |  |  |  |  |  |
| BMI z-score at 12.5^a^ | 0.36 | 0.33 | 0.39 | <0.001 | 0.35 | 0.32 | 0.38 | <0.001 | 0.15 | 0.10 | 0.19 | <0.001 |  |  |  |  |  |
| BMI z-score at 15.5^a^ | 0.36 | 0.33 | 0.40 | <0.001 | 0.35 | 0.32 | 0.39 | <0.001 | 0.11 | 0.06 | 0.16 | <0.001 |  |  |  |  |  |
| BMI (kg/m^2^) at 18^a^ | 0.45 | 0.41 | 0.50 | <0.001 | 0.44 | 0.40 | 0.49 | <0.001 | 0.07 | -0.01 | 0.15 | 0.08 |  |  |  |  |  |
|  | **Model1** | | | | **Model2** | | | | **Model3** | | | | **Model4** | | | |  |
|  | Beta | LCI | UCI | p | Beta | LCI | UCI | p | Beta | LCI | UCI | p | Beta | LCI | UCI | p |  |
| BMI (kg/m^2^) at 24**^b^** | 0.43 | 0.40 | 0.46 | <0.001 | 0.42 | 0.39 | 0.46 | <0.001 | 0.34 | 0.27 | 0.4 | <0.001 | 0.33 | 0.27 | 0.4 | <0.001 |  |
|  | **Model1** | | | | **Model2** | | | | **Model3** | | | |  | | | |  |
| **Males** | Beta | LCI | UCI | p | Beta | LCI | UCI | p | Beta | LCI | UCI | p |  |  |  |  | p (sex interaction)^c^ |
| BMI z-score at 7^a^ | 0.24 | 0.19 | 0.29 | <0.001 | 0.23 | 0.18 | 0.28 | <0.001 | 0.06 | 0.00 | 0.12 | 0.04 |  |  |  |  | 0.82 |
| BMI z-score at 10^a^ | 0.28 | 0.24 | 0.33 | <0.001 | 0.27 | 0.22 | 0.31 | <0.001 | 0.12 | 0.06 | 0.17 | <0.001 |  |  |  |  | 0.97 |
| BMI z-score at 12.5^a^ | 0.30 | 0.25 | 0.34 | <0.001 | 0.28 | 0.24 | 0.33 | <0.001 | 0.14 | 0.08 | 0.19 | <0.001 |  |  |  |  | 0.93 |
| BMI z-score at 15.5^a^ | 0.33 | 0.28 | 0.38 | <0.001 | 0.32 | 0.27 | 0.37 | <0.001 | 0.14 | 0.06 | 0.22 | 0.001 |  |  |  |  | 0.34 |
| BMI (kg/m^2^) at 18^a^ | 0.56 | 0.48 | 0.63 | <0.001 | 0.53 | 0.45 | 0.61 | <0.001 | 0.28 | 0.12 | 0.44 | 0.001 |  |  |  |  | 0.008 |
|  | **Model1** | | | | **Model2** | | | | **Model3** | | | | **Model4** | | | |  |
|  | Beta | LCI | UCI | p | Beta | LCI | UCI | p | Beta | LCI | UCI | p | Beta | LCI | UCI | p |  |
| BMI (kg/m^2^) at 24**^b^** | 0.47 | 0.41 | 0.53 | <0.001 | 0.45 | 0.38 | 0.51 | <0.001 | 0.26 | 0.13 | 0.39 | <0.001 | 0.26 | 0.13 | 0.39 | <0.001 | 0.57 |
| ^a^Model 1 does not adjust for any covariates. Model 2: Model 1 + ethnicity, maternal qualifications, and sexual orientation. Model 3: Model 2 + university attendance. Model 4: Model 3 + BMI at age 24.  **^b^**Model 1 does not adjust for any covariates. Model 2: Model 1 + ethnicity, maternal qualifications, and sexual orientation. Model 3: Model 2 + BMI at ages 7, 10, 12.5, 15.5, and 17.5. Model 4: Model 3 + university attendance and NEET history.  ^c^Sex interaction in fully-adjusted model. | | | | | | | | | | | | | | | | | |

**Table S9: Sex -stratified models: BMI across development and internalized weight stigma at age 31**

|  | **Model1^a^** | | | | **Model2** | | | | **Model3** | | | | **Model4** | | | |  |
| --- | --- | --- | --- | --- | --- | --- | --- | --- | --- | --- | --- | --- | --- | --- | --- | --- | --- |
| **Females** | Beta | LCI | UCI | p | Beta | LCI | UCI | p | Beta | LCI | UCI | p | Beta | LCI | UCI | p |  |
| Negative weight-based comments from parents | 0.58 | 0.49 | 0.68 | <0.001 | 0.57 | 0.48 | 0.66 | <0.001 | 0.16 | 0.05 | 0.27 | 0.003 | 0.13 | 0.03 | 0.24 | 0.01 |  |
| Weight-based teasing: family | 0.48 | 0.37 | 0.58 | <0.001 | 0.47 | 0.37 | 0.57 | <0.001 | 0.17 | 0.06 | 0.28 | 0.002 | 0.17 | 0.07 | 0.27 | 0.001 |  |
| Weight-based teasing at school | 0.47 | 0.37 | 0.57 | <0.001 | 0.45 | 0.35 | 0.55 | <0.001 | 0.17 | 0.08 | 0.27 | <0.001 | 0.06 | -0.04 | 0.15 | 0.24 |  |
| Pressure to lose weight: family | 0.63 | 0.54 | 0.72 | <0.001 | 0.62 | 0.53 | 0.71 | <0.001 | 0.10 | -0.02 | 0.21 | 0.11 | 0.09 | -0.02 | 0.21 | 0.11 |  |
| Pressure to lose weight: friends | 0.48 | 0.39 | 0.58 | <0.001 | 0.47 | 0.38 | 0.57 | <0.001 | 0.06 | -0.05 | 0.16 | 0.29 | 0.06 | -0.04 | 0.16 | 0.26 |  |
| Pressure to lose weight: boys/girls | 0.44 | 0.32 | 0.55 | <0.001 | 0.43 | 0.31 | 0.54 | <0.001 | -0.02 | -0.14 | 0.10 | 0.75 | 0.01 | -0.11 | 0.12 | 0.93 |  |
| Pressure to lose weight: media | 0.51 | 0.43 | 0.60 | <0.001 | 0.51 | 0.43 | 0.60 | <0.001 | 0.16 | 0.07 | 0.25 | 0.001 | 0.17 | 0.09 | 0.26 | <0.001 |  |
|  | **Model1** | | | | **Model2** | | | | **Model3** | | | | **Model4** | | | |  |
| **Males** | Beta | LCI | UCI | p | Beta | LCI | UCI | p | Beta | LCI | UCI | p | Beta | LCI | UCI | p | p (sex interaction)^c^ |
| Negative weight-based comments from parents | 0.55 | 0.38 | 0.71 | <0.001 | 0.50 | 0.34 | 0.66 | <0.001 | 0.17 | 0.00 | 0.34 | 0.05 | 0.15 | -0.01 | 0.32 | 0.07 | 0.16 |
| Weight-based teasing: family | 0.36 | 0.19 | 0.53 | <0.001 | 0.34 | 0.18 | 0.51 | <0.001 | 0.06 | -0.12 | 0.23 | 0.53 | 0.07 | -0.09 | 0.24 | 0.39 | 0.87 |
| Weight-based teasing at school | 0.51 | 0.35 | 0.66 | <0.001 | 0.48 | 0.33 | 0.63 | <0.001 | 0.24 | 0.08 | 0.40 | 0.004 | 0.20 | 0.05 | 0.36 | 0.01 | 0.04 |
| Pressure to lose weight: family | 0.79 | 0.63 | 0.95 | <0.001 | 0.73 | 0.57 | 0.88 | <0.001 | 0.32 | 0.12 | 0.51 | 0.002 | 0.28 | 0.09 | 0.48 | 0.005 | 0.02 |
| Pressure to lose weight: friends | 0.55 | 0.38 | 0.72 | <0.001 | 0.53 | 0.36 | 0.69 | <0.001 | 0.00 | -0.20 | 0.20 | 0.99 | 0.05 | -0.15 | 0.25 | 0.62 | 0.33 |
| Pressure to lose weight: boys/girls | 0.35 | 0.09 | 0.62 | 0.01 | 0.31 | 0.05 | 0.57 | 0.02 | -0.11 | -0.36 | 0.14 | 0.38 | -0.07 | -0.32 | 0.19 | 0.603 | 0.81 |
| Pressure to lose weight: media | 0.57 | 0.37 | 0.77 | <0.001 | 0.51 | 0.32 | 0.71 | <0.001 | 0.14 | -0.06 | 0.34 | 0.17 | 0.14 | -0.06 | 0.35 | 0.17 | 0.52 |
| ^a^Model 1 does not adjust for any covariates. Model 2: Model 1 + ethnicity, maternal qualifications, and sexual orientation. Model 3: Model 2 + BMI at age 12.5 years and other social factors at age 13. Model 4: Model 3 + BMI at age 24.  ^c^Sex interaction in fully-adjusted model. | | | | | | | | | | | | | | | | | |

**Table S10: Sex-stratified models: family, peer, and wider social environment at age 13 and internalized weight stigma at age 31**

**Table S11: Sex-stratified models: bullying victimization at age 8, 10, 12.5, 17.5 and 23 and internalized weight stigma at age 31**

|  | **Model1^a^** | | | | **Model2** | | | | **Model3** | | | | **Model4** | | | |  |
| --- | --- | --- | --- | --- | --- | --- | --- | --- | --- | --- | --- | --- | --- | --- | --- | --- | --- |
| ***Females: any bullying*** | Beta | LCI | UCI | p | Beta | LCI | UCI | p | Beta | LCI | UCI | p | Beta | LCI | UCI | p |  |
| Age 8 | 0.15 | 0.05 | 0.25 | 0.003 | 0.14 | 0.04 | 0.24 | 0.008 | 0.05 | -0.05 | 0.14 | 0.34 | 0.02 | -0.07 | 0.11 | 0.69 |  |
| Age 10 | 0.22 | 0.10 | 0.34 | <0.001 | 0.21 | 0.09 | 0.32 | <0.001 | 0.11 | 0.00 | 0.23 | 0.04 | 0.07 | -0.04 | 0.18 | 0.21 |  |
| Age 12.5 | 0.15 | 0.06 | 0.24 | 0.002 | 0.13 | 0.04 | 0.22 | 0.005 | 0.04 | -0.04 | 0.12 | 0.35 | 0.07 | -0.01 | 0.15 | 0.10 |  |
| Age 17.5 | 0.37 | 0.26 | 0.49 | <0.001 | 0.35 | 0.24 | 0.47 | <0.001 | 0.26 | 0.14 | 0.37 | <0.001 | 0.23 | 0.12 | 0.34 | <0.001 |  |
| Age 23 | 0.44 | 0.33 | 0.55 | <0.001 | 0.41 | 0.30 | 0.52 | <0.001 | 0.26 | 0.16 | 0.36 | <0.001 | 0.26 | 0.16 | 0.36 | <0.001 |  |
|  | **Model1** | | | | **Model2** | | | | **Model3** | | | | **Model4** | | | |  |
| ***Males: any bullying*** | Beta | LCI | UCI | p | Beta | LCI | UCI | p | Beta | LCI | UCI | p | Beta | LCI | UCI | p | p (sex interaction)^c^ |
| Age 8 | 0.20 | 0.08 | 0.33 | 0.002 | 0.17 | 0.05 | 0.30 | 0.006 | 0.10 | -0.02 | 0.22 | 0.11 | 0.08 | -0.05 | 0.20 | 0.23 | 0.48 |
| Age 10 | 0.19 | 0.04 | 0.34 | 0.012 | 0.15 | 0.01 | 0.30 | 0.04 | 0.05 | -0.09 | 0.20 | 0.46 | 0.04 | -0.10 | 0.18 | 0.57 | 0.86 |
| Age 12.5 | 0.08 | -0.04 | 0.20 | 0.218 | 0.06 | -0.06 | 0.18 | 0.32 | -0.04 | -0.16 | 0.08 | 0.50 | -0.01 | -0.13 | 0.10 | 0.82 | 0.30 |
| Age 17.5 | 0.27 | 0.12 | 0.42 | <0.001 | 0.23 | 0.09 | 0.38 | 0.002 | 0.20 | 0.06 | 0.35 | 0.006 | 0.21 | 0.07 | 0.36 | 0.004 | 0.85 |
| Age 23 | 0.32 | 0.12 | 0.52 | 0.002 | 0.26 | 0.07 | 0.45 | 0.008 | 0.19 | 0.01 | 0.37 | 0.04 | 0.19 | 0.01 | 0.37 | 0.041 | 0.69 |
| ^a^Model 1 does not adjust for any covariates. Model 2 adjusts for ethnicity, maternal qualifications and sexual orientation. Model 3: model 2 + BMI at the time and bullying at other timepoints. Model 4: Model 3 + BMI at age 24 years. ^c^Sex interaction in fully-adjusted model. | | | | | | | | | | | | | | | | | |

|  | **Model1^a^** | | | | **Model2** | | | | **Model 3** | | | | **Model 4** | | | |
| --- | --- | --- | --- | --- | --- | --- | --- | --- | --- | --- | --- | --- | --- | --- | --- | --- |
|  | Beta | LCI | UCI | LCI | LCI | LCI | LCI | p | Beta | LCI | UCI | p | Beta | LCI | UCI | p |
| Male | Ref |  |  |  | Ref |  |  |  | Ref |  |  |  |  |  |  |  |
| Female | 0.57 | 0.51 | 0.63 | <0.001 | 0.54 | 0.48 | 0.60 | <0.001 | 0.55 | 0.49 | 0.61 | <0.001 |  |  |  |  |
| White | Ref |  |  |  | Ref |  |  |  | Ref |  |  |  |  |  |  |  |
| Any other ethnicity | 0.00 | -0.15 | 0.16 | 0.98 | 0.00 | -0.13 | 0.14 | 0.96 | 0.00 | -0.14 | 0.14 | 0.99 |  |  |  |  |
| Maternal qualifications: University degree | Ref |  |  |  | Ref |  |  |  | Ref |  |  |  | Ref |  |  |  |
| A-levels/vocational qualifications | 0.20 | 0.11 | 0.29 | <0.001 | 0.20 | 0.11 | 0.29 | <0.001 | 0.12 | 0.04 | 0.20 | 0.003 | 0.11 | 0.03 | 0.19 | 0.005 |
| GCSEs/O-levels or less | 0.32 | 0.24 | 0.41 | <0.001 | 0.32 | 0.24 | 0.41 | <0.001 | 0.19 | 0.11 | 0.26 | <0.001 | 0.17 | 0.10 | 0.25 | <0.001 |
| Sexual orientation: heterosexual | Ref |  |  |  | Ref |  |  |  | Ref |  |  |  | Ref |  |  |  |
| Sexual orientation: any other orientation | 0.27 | 0.19 | 0.35 | <0.001 | 0.28 | 0.20 | 0.37 | <0.001 | 0.17 | 0.09 | 0.24 | <0.001 | 0.17 | 0.09 | 0.24 | <0.001 |
| University attendance by age 30: yes | Ref |  |  |  | Ref |  |  |  | Ref |  |  |  | Ref |  |  |  |
| University attendance by age 30: no | 0.25 | 0.19 | 0.32 | <0.001 | 0.20 | 0.14 | 0.27 | <0.001 | 0.06 | -0.00 | 0.12 | 0.06 | 0.06 | -0.01 | 0.12 | 0.08 |
| NEET age 21-31: never | Ref |  |  |  | Ref |  |  |  | Ref |  |  |  | Ref |  |  |  |
| NEET age 21-31: once | 0.08 | -0.02 | 0.17 | 0.10 | 0.07 | -0.02 | 0.16 | 0.12 | 0.05 | -0.04 | 0.13 | 0.31 | 0.05 | -0.04 | 0.13 | 0.28 |
| NEET age 21-31: 2+ times | 0.34 | 0.22 | 0.46 | <0.001 | 0.29 | 0.17 | 0.42 | <0.001 | 0.17 | 0.06 | 0.29 | 0.003 | 0.19 | 0.08 | 0.31 | 0.001 |
| ^a^Model 1 adjusts for no covariates (sex model) or sex only (all other models). Model 2 adjusts for BMI at age 24 (sex model, ethnicity model), sex and ethnicity (maternal qualifications model), sex, ethnicity, and maternal qualifications (sexual orientation), or sex, ethnicity, maternal qualifications, and sexual orientation (university attendance and NEET history). Model 3: model 2 + BMI at age 24 (all except sex and ethnicity models) or model 2 + BMI at ages 7, 10, 12.5, 15.5, 17.5 years. Model 4: Model 3 + BMI at ages 7, 10, 12.5, 15.5, 17.5 years. | | | | | | | | | | | | | | | | |

**Table S12: Demographic risk factors for internalized weight stigma (11-item WBIS-M) at age 31**

|  | | | | | | | | | | | | | | | | |
| --- | --- | --- | --- | --- | --- | --- | --- | --- | --- | --- | --- | --- | --- | --- | --- | --- |
| ***BMI at age 7 to 17.5 and internalized weight stigma at age 31^a^*** | | | | | | | | | | | | | | | | |
|  | **Model1** | | | | **Model2** | | | | **Model3** | | | |  |  |  |  |
|  | Beta | LCI | UCI | p | Beta | LCI | UCI | p | Beta | LCI | UCI | p |  |  |  |  |
| BMI z-score at 7 | 0.27 | 0.24 | 0.30 | <0.001 | 0.26 | 0.23 | 0.29 | <0.001 | 0.06 | 0.02 | 0.09 | 0.001 |  |  |  |  |
| BMI z-score at 10 | 0.30 | 0.27 | 0.32 | <0.001 | 0.29 | 0.26 | 0.31 | <0.001 | 0.10 | 0.07 | 0.13 | <0.001 |  |  |  |  |
| BMI z-score at 12.5 | 0.32 | 0.30 | 0.35 | <0.001 | 0.31 | 0.29 | 0.33 | <0.001 | 0.12 | 0.09 | 0.15 | <0.001 |  |  |  |  |
| BMI z-score at 15.5 | 0.34 | 0.31 | 0.36 | <0.001 | 0.33 | 0.30 | 0.35 | <0.001 | 0.09 | 0.06 | 0.13 | <0.001 |  |  |  |  |
| BMI at 17.5 (per 5kg/m^2^) | 0.46 | 0.42 | 0.49 | <0.001 | 0.44 | 0.41 | 0.48 | <0.001 | 0.11 | 0.05 | 0.18 | <0.001 |  |  |  |  |
| ***^a^***Model 1 adjusts for sex. Model 2: Model 1 + ethnicity, maternal qualifications, and sexual orientation. Model 3: Model 2 + BMI at age 24. | | | | | | | | | | | | | | | | |
| ***BMI at age 24 and internalized weight stigma at age 31^b^*** | | | | | | | | | | | | | | | | |
|  | **Model1** | | | | **Model2** | | | | **Model3** | | | | **Model4** | | | |
|  | Beta | LCI | UCI | p | Beta | LCI | UCI | p | Beta | LCI | UCI | p | Beta | LCI | UCI | p |
| BMI at 24 (per 5kg/m^2^) | 0.44 | 0.41 | 0.46 | <0.001 | 0.42 | 0.39 | 0.45 | <0.001 | 0.32 | 0.27 | 0.38 | <0.001 | 0.32 | 0.26 | 0.37 | <0.001 |
| **^b^**Model 1 adjusts for sex. Model 2: Model 1 + ethnicity, maternal qualifications, and sexual orientation. Model 3: Model 2 + BMI at ages 7, 10, 12.5, 15.5, and 17.5. Model 4: Model 3 + university attendance and NEET history. | | | | | | | | | | | | | | | | |

**Table S13: BMI across development and internalized weight stigma (11-item WBIS-M) at age 31**

|  | **Model1^a^** | | | | **Model2** | | | | **Model3** | | | | **Model4** | | | |
| --- | --- | --- | --- | --- | --- | --- | --- | --- | --- | --- | --- | --- | --- | --- | --- | --- |
|  | Beta | LCI | UCI | p | Beta | LCI | UCI | p | Beta | LCI | UCI | p | Beta | LCI | UCI | p |
| Negative weight-based comments from parents | 0.57 | 0.49 | 0.64 | <0.001 | 0.55 | 0.47 | 0.62 | <0.001 | 0.18 | 0.09 | 0.26 | <0.001 | 0.14 | 0.06 | 0.22 | 0.001 |
| Weight-based teasing: family | 0.44 | 0.36 | 0.52 | <0.001 | 0.43 | 0.35 | 0.50 | <0.001 | 0.14 | 0.06 | 0.22 | 0.001 | 0.14 | 0.07 | 0.22 | <0.001 |
| Weight-based teasing at school | 0.46 | 0.38 | 0.54 | <0.001 | 0.44 | 0.36 | 0.52 | <0.001 | 0.18 | 0.10 | 0.26 | <0.001 | 0.09 | 0.01 | 0.17 | 0.03 |
| Pressure to lose weight: family | 0.65 | 0.57 | 0.72 | <0.001 | 0.63 | 0.55 | 0.71 | <0.001 | 0.14 | 0.04 | 0.23 | 0.006 | 0.13 | 0.04 | 0.23 | 0.006 |
| Pressure to lose weight: friends | 0.47 | 0.39 | 0.56 | <0.001 | 0.46 | 0.38 | 0.54 | <0.001 | 0.03 | -0.06 | 0.12 | 0.53 | 0.04 | -0.05 | 0.13 | 0.34 |
| Pressure to lose weight: boys/girls | 0.38 | 0.27 | 0.49 | <0.001 | 0.37 | 0.26 | 0.47 | <0.001 | -0.06 | -0.16 | 0.05 | 0.31 | -0.03 | -0.13 | 0.07 | 0.58 |
| Pressure to lose weight: media | 0.53 | 0.45 | 0.60 | <0.001 | 0.51 | 0.44 | 0.59 | <0.001 | 0.19 | 0.11 | 0.27 | <0.001 | 0.19 | 0.11 | 0.27 | <0.001 |
| **^a^**Model 1 adjusts for sex. Model 2: Model 1 + ethnicity, maternal qualifications, and sexual orientation. Model 3: Model 2 + BMI at age 12.5 years and other age 13 factors. Model 4: Model 3 + BMI at age 24 years. | | | | | | | | | | | | | | | | |

**Table S14: Family, peer, and wider social environment at age 13 years and internalized weight stigma (11-item WBIS-M) at age 31**

|  | **Model1^a^** | | | | **Model2** | | | | **Model3** | | | | **Model4** | | | |
| --- | --- | --- | --- | --- | --- | --- | --- | --- | --- | --- | --- | --- | --- | --- | --- | --- |
| ***Any bullying*** | Beta | LCI | UCI | p | Beta | LCI | UCI | p | Beta | LCI | UCI | p | Beta | LCI | UCI | p |
| Age 8 | 0.13 | 0.06 | 0.21 | <0.001 | 0.12 | 0.04 | 0.19 | 0.002 | 0.04 | -0.03 | 0.10 | 0.29 | 0.01 | -0.05 | 0.08 | 0.69 |
| Age 10 | 0.19 | 0.11 | 0.27 | <0.001 | 0.17 | 0.09 | 0.25 | <0.001 | 0.08 | 0.00 | 0.16 | 0.05 | 0.05 | -0.02 | 0.12 | 0.18 |
| Age 12.5 | 0.13 | 0.06 | 0.19 | <0.001 | 0.11 | 0.04 | 0.18 | 0.001 | 0.02 | -0.05 | 0.08 | 0.57 | 0.04 | -0.02 | 0.10 | 0.20 |
| Age 17.5 | 0.19 | 0.11 | 0.27 | <0.001 | 0.18 | 0.10 | 0.25 | <0.001 | 0.30 | 0.21 | 0.38 | <0.001 | 0.28 | 0.19 | 0.36 | <0.001 |
| Age 23 | 0.26 | 0.17 | 0.35 | <0.001 | 0.25 | 0.16 | 0.34 | <0.001 | 0.40 | 0.31 | 0.49 | <0.001 | 0.36 | 0.27 | 0.45 | <0.001 |
| ^a^Model 1 adjusts for sex. Model 2 adjusts for sex, ethnicity, maternal qualifications, and sexual orientation. Model 3: model 2 + BMI at the time and bullying at other timepoints. Model 4: Model 3 + BMI at age 24 years. | | | | | | | | | | | | | | | | |

**Table S15: Bullying victimization at age 8, 10, 12.5, 17.5 and 23 and internalized weight stigma (11-item WBIS-M) at age 31**

|  | **Model1^a^** | | | | **Model2** | | | | **Model 3** | | | | **Model 4** | | | |
| --- | --- | --- | --- | --- | --- | --- | --- | --- | --- | --- | --- | --- | --- | --- | --- | --- |
|  | Beta | LCI | UCI | p | Beta | LCI | UCI | p | Beta | LCI | UCI | p | Beta | LCI | UCI | p |
| Male | Ref |  |  |  | Ref |  |  |  | Ref |  |  |  |  |  |  |  |
| Female | 0.49 | 0.39 | 0.58 | <0.001 | 0.49 | 0.41 | 0.57 | <0.001 | 0.50 | 0.42 | 0.58 | <0.001 |  |  |  |  |
| White | Ref |  |  |  | Ref |  |  |  | Ref |  |  |  |  |  |  |  |
| Any other ethnicity | -0.05 | -0.30 | 0.20 | 0.70 | -0.04 | -0.25 | 0.18 | 0.75 | -0.03 | -0.25 | 0.18 | 0.76 |  |  |  |  |
| Maternal qualifications: University degree | Ref |  |  |  | Ref |  |  |  | Ref |  |  |  | Ref |  |  |  |
| A-levels/vocational qualifications | 0.16 | 0.03 | 0.28 | 0.01 | 0.16 | 0.03 | 0.28 | 0.01 | 0.05 | -0.06 | 0.16 | 0.35 | 0.05 | -0.06 | 0.15 | 0.38 |
| GCSEs/O-levels or less | 0.23 | 0.11 | 0.35 | <0.001 | 0.23 | 0.11 | 0.35 | <0.001 | 0.10 | 0.00 | 0.21 | 0.05 | 0.10 | 0.00 | 0.20 | 0.06 |
| Sexual orientation: heterosexual | Ref |  |  |  | Ref |  |  |  | Ref |  |  |  | Ref |  |  |  |
| Sexual orientation: any other orientation | 0.23 | 0.11 | 0.36 | <0.001 | 0.24 | 0.12 | 0.37 | <0.001 | 0.14 | 0.03 | 0.25 | 0.01 | 0.14 | 0.03 | 0.24 | 0.01 |
| University attendance by age 30: yes | Ref |  |  |  | Ref |  |  |  | Ref |  |  |  | Ref |  |  |  |
| University attendance by age 30: no | 0.22 | 0.11 | 0.33 | <0.001 | 0.18 | 0.06 | 0.29 | 0.002 | 0.03 | -0.07 | 0.13 | 0.54 | 0.03 | -0.07 | 0.14 | 0.50 |
| NEET age 21-31: never | Ref |  |  |  | Ref |  |  |  | Ref |  |  |  | Ref |  |  |  |
| NEET age 21-31: once | 0.07 | -0.09 | 0.24 | 0.39 | 0.06 | -0.10 | 0.23 | 0.44 | 0.03 | -0.11 | 0.18 | 0.67 | 0.04 | -0.11 | 0.18 | 0.60 |
| NEET age 21-31: 2+ times | 0.35 | 0.01 | 0.70 | 0.04 | 0.29 | -0.05 | 0.63 | 0.10 | 0.10 | -0.20 | 0.40 | 0.51 | 0.11 | -0.19 | 0.40 | 0.49 |
| ^a^Model 1 adjusts for no covariates (sex model) or sex only (all other models). Model 2 adjusts for BMI at age 24 (sex model, ethnicity model), sex and ethnicity (maternal qualifications model), sex, ethnicity, and maternal qualifications (sexual orientation), or sex, ethnicity, maternal qualifications, and sexual orientation (university attendance and NEET history). Model 3: model 2 + BMI at age 24 (all except sex and ethnicity models) or model 2 + BMI at ages 7, 10, 12.5, 15.5, 17.5 years. Model 4: Model 3 + BMI at ages 7, 10, 12.5, 15.5, 17.5 years. | | | | | | | | | | | | | | | | |

**Table S16: Demographic risk factors for internalized weight stigma (complete-case analysis) at age 31**

|  | | | | | | | | | | | | | | | | |
| --- | --- | --- | --- | --- | --- | --- | --- | --- | --- | --- | --- | --- | --- | --- | --- | --- |
| ***BMI at age 7 to 17.5 and internalized weight stigma at age 31^a^*** | | | | | | | | | | | | | | | | |
|  | **Model1** | | | | **Model2** | | | | **Model3** | | | |  |  |  |  |
|  | Beta | LCI | UCI | p | Beta | LCI | UCI | p | Beta | LCI | UCI | p |  |  |  |  |
| BMI z-score at 7 | 0.26 | 0.22 | 0.3 | <0.001 | 0.25 | 0.21 | 0.29 | <0.001 | 0.03 | -0.01 | 0.07 | 0.19 |  |  |  |  |
| BMI z-score at 10 | 0.27 | 0.24 | 0.31 | <0.001 | 0.26 | 0.23 | 0.30 | <0.001 | 0.06 | 0.02 | 0.10 | 0.002 |  |  |  |  |
| BMI z-score at 12.5 | 0.29 | 0.26 | 0.32 | <0.001 | 0.28 | 0.25 | 0.31 | <0.001 | 0.06 | 0.02 | 0.10 | 0.002 |  |  |  |  |
| BMI z-score at 15.5 | 0.32 | 0.28 | 0.35 | <0.001 | 0.31 | 0.27 | 0.34 | <0.001 | 0.05 | 0.01 | 0.10 | 0.02 |  |  |  |  |
| BMI at 17.5 (per 5kg/m^2^) | 0.47 | 0.42 | 0.51 | <0.001 | 0.46 | 0.41 | 0.50 | <0.001 | 0.08 | 0.01 | 0.15 | 0.02 |  |  |  |  |
| ***^a^***Model 1 adjusts for sex. Model 2: Model 1 + ethnicity, maternal qualifications, and sexual orientation. Model 3: Model 2 + BMI at age 24. | | | | | | | | | | | | | | | | |
| ***BMI at age 24 and internalized weight stigma at age 31^b^*** | | | | | | | | | | | | | | | | |
|  | **Model1** | | | | **Model2** | | | | **Model3** | | | | **Model4** | | | |
|  | Beta | LCI | UCI | p | Beta | LCI | UCI | p | Beta | LCI | UCI | p | Beta | LCI | UCI | p |
| BMI at 24 (per 5kg/m^2^) | 0.47 | 0.41 | 0.52 | <0.001 | 0.46 | 0.40 | 0.52 | <0.001 | 0.42 | 0.32 | 0.51 | <0.001 | 0.41 | 0.32 | 0.51 | <0.001 |
| **^b^**Model 1 adjusts for sex. Model 2: Model 1 + ethnicity, maternal qualifications, and sexual orientation. Model 3: Model 2 + BMI at ages 7, 10, 12.5, 15.5, and 17.5. Model 4: Model 3 + university attendance and NEET history. | | | | | | | | | | | | | | | | |

**Table S17: BMI across development and internalized weight stigma (complete-case analysis) at age 31**

|  | **Model1^a^** | | | | **Model2** | | | | **Model3** | | | | **Model4** | | | |
| --- | --- | --- | --- | --- | --- | --- | --- | --- | --- | --- | --- | --- | --- | --- | --- | --- |
|  | Beta | LCI | UCI | p | Beta | LCI | UCI | p | Beta | LCI | UCI | p | Beta | LCI | UCI | p |
| Negative weight-based comments from parents | 0.56 | 0.46 | 0.66 | <0.001 | 0.54 | 0.45 | 0.64 | <0.001 | 0.20 | 0.09 | 0.31 | <0.001 | 0.16 | 0.06 | 0.26 | 0.002 |
| Weight-based teasing: family | 0.42 | 0.32 | 0.52 | <0.001 | 0.40 | 0.30 | 0.50 | <0.001 | 0.11 | 0.01 | 0.22 | 0.04 | 0.12 | 0.03 | 0.22 | 0.01 |
| Weight-based teasing at school | 0.39 | 0.29 | 0.49 | <0.001 | 0.37 | 0.27 | 0.47 | <0.001 | 0.14 | 0.04 | 0.24 | 0.008 | 0.05 | -0.05 | 0.15 | 0.30 |
| Pressure to lose weight: family | 0.64 | 0.54 | 0.74 | <0.001 | 0.63 | 0.52 | 0.73 | <0.001 | 0.19 | 0.07 | 0.32 | 0.002 | 0.17 | 0.06 | 0.29 | 0.004 |
| Pressure to lose weight: friends | 0.43 | 0.32 | 0.53 | <0.001 | 0.42 | 0.31 | 0.52 | <0.001 | -0.04 | -0.15 | 0.08 | 0.55 | -0.01 | -0.12 | 0.10 | 0.84 |
| Pressure to lose weight: boys/girls | 0.43 | 0.30 | 0.56 | <0.001 | 0.42 | 0.29 | 0.54 | <0.001 | 0.02 | -0.11 | 0.16 | 0.72 | 0.05 | -0.08 | 0.17 | 0.45 |
| Pressure to lose weight: media | 0.48 | 0.39 | 0.58 | <0.001 | 0.47 | 0.37 | 0.56 | <0.001 | 0.15 | 0.05 | 0.26 | 0.003 | 0.16 | 0.06 | 0.25 | 0.001 |
| **^a^**Model 1 adjusts for sex. Model 2: Model 1 + ethnicity, maternal qualifications, and sexual orientation. Model 3: Model 2 + BMI at age 12.5 years and other age 13 factors. Model 4: Model 3 + BMI at age 24 years. | | | | | | | | | | | | | | | | |

**Table S18: Family, peer, and wider social environment at age 13 years and internalized weight stigma (complete-case analysis) at age 31**

|  | **Model1^a^** | | | | **Model2** | | | | **Model3** | | | | **Model4** | | | |
| --- | --- | --- | --- | --- | --- | --- | --- | --- | --- | --- | --- | --- | --- | --- | --- | --- |
| ***Any bullying*** | Beta | LCI | UCI | p | Beta | LCI | UCI | p | Beta | LCI | UCI | p | Beta | LCI | UCI | p |
| Age 8 | 0.12 | 0.03 | 0.21 | 0.007 | 0.11 | 0.02 | 0.20 | 0.01 | 0.07 | -0.02 | 0.15 | 0.13 | 0.03 | -0.05 | 0.11 | 0.48 |
| Age 10 | 0.19 | 0.10 | 0.29 | <0.001 | 0.18 | 0.08 | 0.27 | <0.001 | 0.11 | 0.02 | 0.20 | 0.02 | 0.09 | 0.00 | 0.18 | 0.04 |
| Age 12.5 | 0.12 | 0.04 | 0.20 | 0.002 | 0.10 | 0.02 | 0.18 | 0.01 | 0.03 | -0.05 | 0.10 | 0.46 | 0.05 | -0.02 | 0.12 | 0.19 |
| Age 17.5 | 0.30 | 0.20 | 0.39 | <0.001 | 0.28 | 0.18 | 0.37 | <0.001 | 0.23 | 0.14 | 0.32 | <0.001 | 0.22 | 0.13 | 0.30 | <0.001 |
| Age 23 | 0.42 | 0.31 | 0.52 | <0.001 | 0.38 | 0.27 | 0.49 | <0.001 | 0.29 | 0.20 | 0.39 | <0.001 | 0.29 | 0.20 | 0.39 | <0.001 |
| ^a^Model 1 adjusts for sex. Model 2 adjusts for sex, ethnicity, maternal qualifications, and sexual orientation. Model 3: model 2 + BMI at the time and bullying at other timepoints. Model 4: Model 3 + BMI at age 24 years. | | | | | | | | | | | | | | | | |

**Table S19: Bullying victimization at age 8, 10, 12.5, 17.5 and 23 and internalized weight stigma (complete-case) at age 31**

1. Pearl, R. L. & Puhl, R. M. Measuring internalized weight attitudes across body weight categories: validation of  the modified weight bias internalization scale. *Body Image* **11**, 89–92 (2014).

2. Hilbert, A. *et al.* Weight bias internalization scale: Psychometric properties and population norms. *PLoS One* (2014) doi:10.1371/journal.pone.0086303.

3. Lee, M. S. & Dedrick, R. F. Weight Bias Internalization Scale: Psychometric properties using alternative weight status classification approaches. *Body Image* **17**, 25–29 (2016).

4. Fraser, A. *et al.* Cohort profile: The avon longitudinal study of parents and children: ALSPAC mothers cohort. *Int J Epidemiol* (2013) doi:10.1093/ije/dys066.

5. Boyd, A. *et al.* Cohort profile: The ’Children of the 90s’-The index offspring of the avon longitudinal study of parents and children. *Int J Epidemiol* (2013) doi:10.1093/ije/dys064.

6. Northstone, K. *et al.* The Avon Longitudinal Study of Parents and Children (ALSPAC): an update on the enrolled sample of index children in 2019 [version 1; peer review: 2 approved]. *Wellcome Open Res* **4**, (2019).

7. Morris, T. T., Dorling, D., Davies, N. M. & Davey Smith, G. Associations between school enjoyment at age 6 and later educational achievement: evidence from a UK cohort study. *NPJ Sci Learn* **6**, 18 (2021).

8. Harris, P. A. *et al.* Research electronic data capture (REDCap)—A metadata-driven methodology and workflow process for providing translational research informatics support. *J Biomed Inform* **42**, 377–381 (2009).
